# Iron(0)‐Mediated Stereoselective (3+2)‐Cycloaddition of Thiochalcones via a Diradical Intermediate

**DOI:** 10.1002/chem.202001412

**Published:** 2020-08-10

**Authors:** Philipp Buday, Phillip Seeber, Clara Zens, Hassan Abul‐Futouh, Helmar Görls, Stefanie Gräfe, Piotr Matczak, Stephan Kupfer, Wolfgang Weigand, Grzegorz Mloston

**Affiliations:** ^1^ Institute of Inorganic and Analytical Chemistry Friedrich Schiller University Jena Humboldtstrasse 8 07743 Jena Germany; ^2^ Institute of Physical Chemistry and Abbe Center of Photonics Friedrich Schiller University Jena Helmholtzweg 4 07743 Jena Germany; ^3^ Faculty of Pharmacy Al-Zaytoonah University of Jordan P.O.Box 130 Amman 11733 Jordan; ^4^ Faculty of Chemistry University of Lodz Tamka 12 91403 Łódź Poland

**Keywords:** cycloaddition, cyclopentenes, diastereoselectivity, iron carbonyls, quantum chemistry

## Abstract

Reactions of α,β‐unsaturated aromatic thioketones **1** (thiochalcones) with Fe_3_(CO)_12_ leading to η^4^‐1‐thia‐1,3‐diene iron tricarbonyl complexes **2**, [FeFe] hydrogenase mimics **3**, and the thiopyrane adduct **4** are described. Obtained products have been characterized by X‐ray crystallography and by computational methods. Completely regio‐ and diastereoselective formation of the five‐membered ring system in products **3**, containing four stereogenic centers, can be explained by an unprecedented, stepwise (3+2)‐cycloaddition of two thiochalcone molecules mediated by Fe_3_(CO)_12_. Quantum chemical calculations aimed at elucidation of the reaction mechanism, suggest that the formal (3+2)‐cycloaddition proceeds via sequential intramolecular radical transfer events upon homolytic cleavage of one carbon‐sulfur bond leading to a diradical intermediate.

## Graphical abstract

**Making the rounds**: A series of new cyclopentenes was obtained from thiochalcones via a Fe^0^‐mediated formal (3+2)‐cycloaddition. Reactions occurred diastereoselectively and the postulated reaction mechanism was elucidated by a combined experimental and theoretical approach.

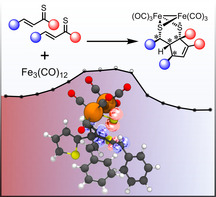

In recent four decades, rapid progress in exploring thiocarbonyl compounds such as thioketones and thiochalcones has been observed.^1^ On the one hand, aromatic thioketones were demonstrated to act as highly reactive dipolarophiles, thus R. Huisgen proposed for them the term “superdipolarophiles” based on results of kinetic studies with such 1,3‐dipoles as nitrones,2a diazomethanes2b and thiocarbonyl *S*‐methanides2c (Scheme 1, top). On the other hand, the observed high reactivity of thioketones in hetero‐Diels–Alder reactions led to the term “superdienophiles” coined by J. Sauer.^3^ Therefore, thiochalcones, easily available by thionation of chalcones with Lawesson's reagent, form a unique class of α,β‐unsaturated aromatic thioketones which are able to react as heterodienes with olefinic and acetylenic dienophiles yielding six‐membered thiopyrane derivatives (Scheme 1, middle).^4^ Furthermore, thiochalcones were also described as reactive dienophiles towards in situ generated α‐nitrosoalkenes5a, 5b and chiral enamines.5c Unexpectedly, thiochalcones reacted as C=S dipolarophiles in (3+2)‐cycloadditions with electron deficient fluorinated nitrile imines derived from trifluoroacetonitrile.^6^

**Scheme 1:**
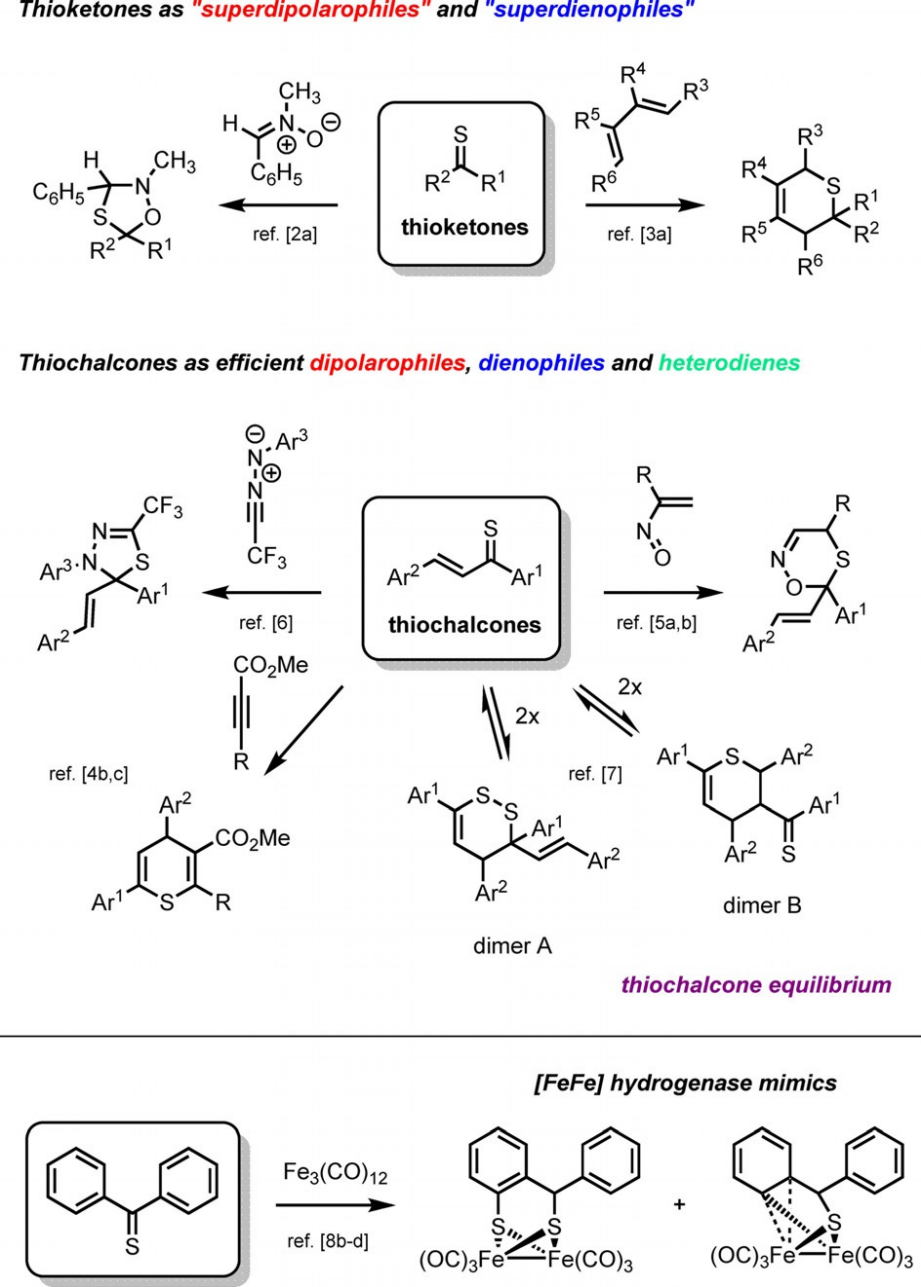
Reactivity of thioketones (top), reactivity of thiochalcones (middle) and application of thioketones (e.g. thiobenzophenone) for preparation of [FeFe] hydrogenase mimics (bottom).

Characteristically, in solution, during chromatographic workup, thiochalcones form an equilibrium mixture of the monomeric and two dimeric species, namely 3,4‐dihydro‐1,2‐dithiin (dimer A) as well as 3,4‐dihydro‐2*H*‐thiopyran (dimer B) derivatives, according to the rules of hetero‐Diels–Alder chemistry (Scheme 1, middle).^7^ On preparative scale, an efficient separation of these three species is practically unfeasible by chromatographic methods, and therefore they are isolated as so‐called “thiochalcone fraction”.5b Depending on the reaction conditions, for example, solvent, temperature, catalyst and the reagent applied for their conversion, thiochalcones can react either as monomers or dimers, thus yielding a variety of different products. For that reason, thiochalcones should be considered as versatile and valuable but also “capricious” building blocks in the organic chemistry of sulfur.

Along with the cycloaddition chemistry described above, another research field focusses on exploring thioketones in coordination and organometallic chemistry, particularly in the synthesis of [FeFe] hydrogenase mimics.^8^ In recent decades, a great number of such [FeFe] hydrogenase model complexes has been reported.^9^ In this respect, our earlier publications describe the preparation of [2Fe1S] and [2Fe2S] clusters, consi‐ dered as mimics of [FeFe] hydrogenases, via a multi‐step re‐ action of aromatic thioketones with Fe_3_(CO)_12_ (Scheme 1, bottom).8b, 8c, 8d

In contrast to aromatic thioketones, the exploration of thiochalcones in coordination and organometallic chemistry is scarcely reported. For example, unlike extensively studied reactions of 1‐aza‐1,3‐dienes and 1‐oxa‐1,3‐dienes with iron carbonyls,^10^ analogous reactions with 1‐thia‐1,3‐dienes, e.g. monomeric thiochalcones, are almost undiscovered.^11^

Stimulated by the aforementioned observations on aromatic thioketones, we investigated the reactions of Fe_3_(CO)_12_ with diverse aryl‐, hetaryl‐ and ferrocenyl‐functionalized thiochalcones in a combined experimental and theoretical study. In extension of the synthetic work, quantum chemical calculations were carried out to unravel the reaction pathway leading to unexpe‐ cted products.

The first series of experiments was performed starting with Fe_3_(CO)_12_ and thiochalcone **1 a** (Scheme 2) in boiling THF. As the accessible reaction pathways crucially depend on the initial configuration of the thiochalcones, a precise knowledge of the reactants at the molecular level is indispensable. Therefore, LNO‐CCSD(T)^12^ calculations were performed to elucidate the structural and electronic properties of four conceivable confi‐ gurations of **1 a**, that is, a pair of *E* (**E_A_** and **E_B_**) and *Z* (**Z_A_** and **Z_B_**) isomers (Scheme 2, top). The formation and reactivity of dimers A and B (Scheme 2) has already been studied by theoretical methods.5b The computational results for **1 a** clearly indicate the thermodynamic preference of the *E* isomers, of which **E_A_** is as much as 6.8 kJ mol^−1^ more stable than **E_B_**.

**Scheme 2:**
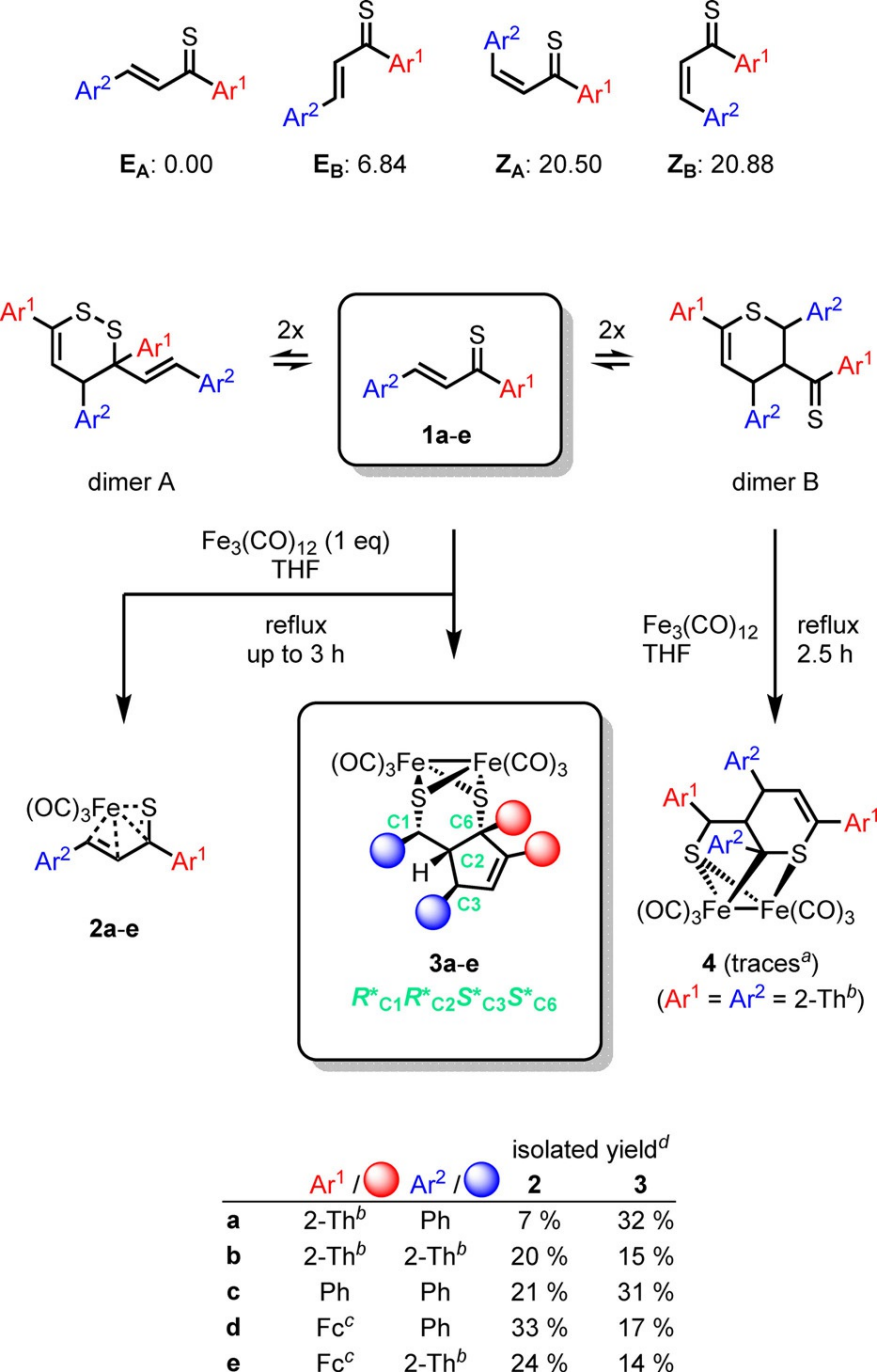
Reactions of thiochalcones **1 a**–**e** with Fe_3_(CO)_12_ resulting in mono and cyclic dinuclear iron complexes **2 a**–**e** and **3 a**–**e**, respectively, as well as in thiopyran complex **4** (isolated for substituents Ar^1^=Ar^2^=2‐Th^[b]^, only). Relative Gibbs energies of **1 a** (in kJ mol^−1^) were obtained at the LNO‐CCSD(T)/cc‐pVQZ level of theory. [a] Unexpectedly, a second experiment with another sample of **1 b** resulted in a better yield of **4**. [b] 2‐Th=thien‐2‐yl. [c] Fc=ferrocenyl. [d] Calculated for the reactions of **1 a**–**e** with Fe_3_(CO)_12_ in 1:1 ratio (in the case of 3:1 ratio **2 a** was obtained in 22 % yield and **3 a** in 6 % yield).

Both reagents, the thiochalcone **1 a** and Fe_3_(CO)_12_, were applied in a ratio of 3:1. After 1 h, **1 a** was completely consumed and chromatographic separation of the oily crude material afforded a major fraction as a red solid. The ^1^H NMR spectrum revealed the presence of two doublets with ^3^ *J*_H,H_=9.4 Hz localized at 6.91 ppm and 3.34 ppm, respectively. The molecular formula C_16_H_10_FeO_3_S_2_ was confirmed by elemental analysis and EI‐MS methods. These data suggest that the structure of the obtained product can be postulated as the η^4^‐1‐thia‐1,3‐diene iron tricarbonyl complex **2 a** (22 %, Scheme 2). Finally, this structure was unambiguously confirmed by X‐ray diffraction analysis (Figure S5). The formation of **2 a** can be explained by interaction of thiochalcone **1 a** with Fe_3_(CO)_12_ in a similar fa‐ shion as previously described for analogous (η^4^‐diene)Fe(CO)_3_ complexes.10b Notably, the structure of **2 a** is comparable to those reported for tricarbonyl iron 1:1 complexes derived from α,β‐unsaturated thioamides and thioesters.^11^

During the chromatographic separation, a more polar fraction was also isolated as minor product **3 a** (6 %, Scheme 2). In contrast to **2 a**, the ^1^H NMR spectrum of this product was more complex. For example, two doublets were found at 5.98 ppm and 3.85 ppm (^3^ *J*_H,H_=2.1 Hz and 4.4 Hz, respectively). Moreover, two doublets of doublets appeared at 4.08 ppm as well as at 3.56 ppm. Three *H*C(sp^3^) atoms were identified in COSY and HSQC NMR spectra at 4.08 ppm, 3.85 ppm and 3.56 ppm. The elemental analysis of that fraction allowed to establish the molecular formula as C_32_H_20_Fe_2_O_6_S_4_ which was also confirmed in the EI‐MS by virtue of *m*/*z=*647 [*M*‐2 CO]^+^. The X‐ray analysis of **3 a** revealed its novel, yet rather unexpected structure, typical of a [FeFe] hydrogenase mimic (Figure 1). The molecular structure of **3 a**, determined by X‐ray crystallography, disclosed its relative R*_C1_ R*_C2_ S*_C3_ S*_C6_ configuration. Structurally, complex **3 a** features a five‐membered cyclopentene ring coordinated via two thiolato sulfur atoms to the Fe_2_(CO)_6_ moiety. It was formed in a completely regio‐ and diastereoselective manner, demonstrated by the presence of only one set of signals found in both the ^1^H and ^13^C{^1^H} NMR spectra of the isolated fraction.

**Figure 1:**
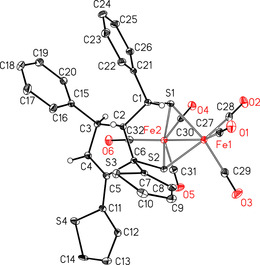
Molecular structure and atom labeling scheme of **3 a**. The ellipsoids represent a probability of 30 %, H atoms are shown with arbitrary radii. Selected bond lengths (in Å): Fe(1)−Fe(2) 2.5148(4), Fe(1)−S(1) 2.2510(5), Fe(1)−S(2) 2.2615(5), Fe(2)−S(1) 2.2790(5), Fe(2)−S(2) 2.2406(5), C(4)−C(5) 1.340(2); angles (in °): S(1)‐Fe(1)‐S(2) 84.009(17), S(1)‐Fe(1)‐Fe(2) 56.811(14), S(2)‐Fe(1)‐Fe(2) 55.649(14), Fe(1)‐S(1)‐Fe(2) 67.439(15), Fe(2)‐S(2)‐Fe(1) 67.914(15).

In order to elucidate the reaction mechanism leading to the diastereoselective formation of **3 a**, quantum chemical calculations were performed (Figure 2 A). A set of 16 adduct states, originating from the coordination of the four possible isomers of **1 a** (**E_A_**, **E_B_**, **Z_A_** and **Z_B_**) at the Fe_2_(CO)_6_ moiety, was consi‐ dered. Two of these adduct states are sterically conceivable to yield **3 a** diastereoselectively (Figure S1 and Table S1). These two states originate from the most stable thiochalcone isomer **E_A_**, as well as from the combination of one **E_A_** and one **Z_B_**. However, to reduce the computational effort, and due to the unfavorable thermodynamic properties of the *Z* isomers, only two **E_A_** isomers were used to study the reaction pathway. The reaction path connecting the closed‐shell optimized structures of the **E_A_**‐**E_A_** adduct, denoted **A(E_A_E_A_)**, as well as of the **E_A_**‐**E_A_** product, denoted **P(E_A_E_A_)**, was approximated based on the climbing image nudge elastic band (CI‐NEB^13^) method (Figure 2 A).

**Figure 2:**
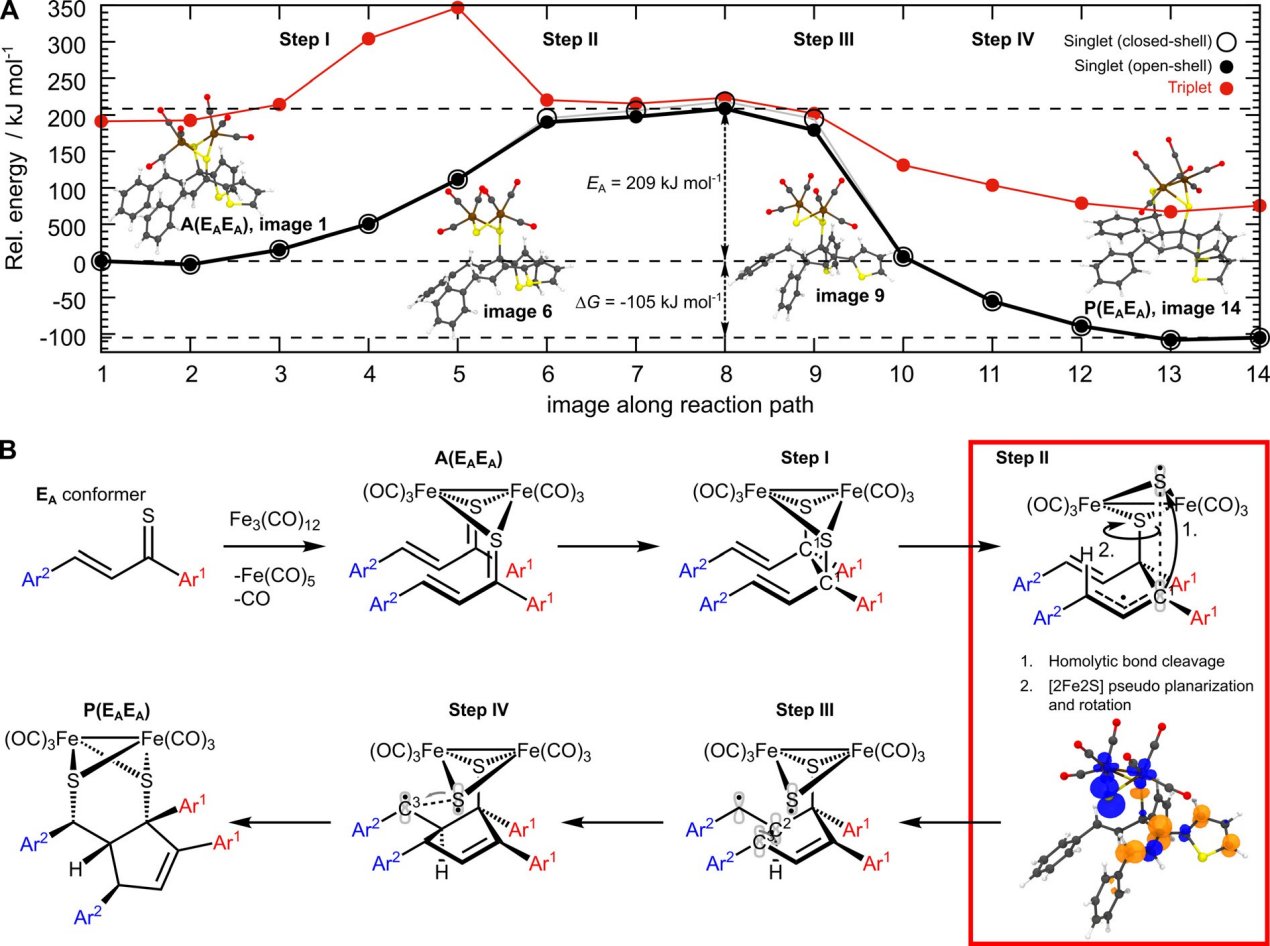
**A**) Reaction profile for the formation of **3 a**, connecting adduct **A(E_A_E_A_)** and product **P(E_A_E_A_)**, calculated at the TPSSh/def2‐SVP level of theory along the closed‐shell singlet path obtained by the CI‐NEB method. **B**) Proposed reaction mechanism and spin density upon homolytic cleavage of one carbon–sulfur bond.

Images along the closed‐shell singlet ground state reaction path were optimized at the TPSSh^14^/def2‐SVP^15^ level of theory; additional open‐shell singlet as well as triplet single point calculations were also performed. Initially, an instantaneous carbon–carbon bond formation (C^1^−C^1^) was observed (Figure 2 B, step I). Subsequently, homolytic cleavage of one carbon–sulfur bond proceeded, as indicated by the energetically favored open‐shell singlet solution. This C−S bond breaking is accompanied by a partial planarization of the [2Fe2S] cluster from its initial butterfly structure, yielding a diradical intermediate with one unpaired electron mainly localized at the sulfur atom and the other electron delocalized along the π*‐orbital of the C^1^‐C^2^‐C^3^ fragment, respectively (step II in Figure 2 B). The localization of the two unpaired electrons is illu‐ strated by the spin density in the vicinity of the transition state. Consequently, an activation energy of 209 kJ mol^−1^ is predicted, which is 10 kJ mol^−1^ lower than for a heterolytic C−S bond cleavage. In addition to the C−S cleavage and the [2Fe2S] planarization, this energy is necessary to facilitate the rotation of the [2Fe2S] cluster around the remaining C−S bond hindered by the axial hydrogen atom *H*C^3^. Surprisingly, this barrier is neither reduced considering implicit solvent effects (THF) nor considering an open‐shell (triplet) path (Figure S2 B‐C). However, a pronounced stabilization of the opened‐shell singlet solutions is expected along its optimized reaction path. Unfortunately, such open‐shell singlet path, connecting the closed‐shell adduct and product states, cannot be obtained as the corresponding method has not (yet) been implemented in the software packages available. Furthermore, a pronounced stabilization of step II may be anticipated by explicit interaction of **3 a** with solvent molecules. As for the interaction between two thiochalcone molecules forming **3 a**, the singly‐occupied π*‐orbital of the C^1^‐C^2^‐C^3^ fragment of the first molecule overlaps with the π*‐orbital of the C^2^−C^3^ fragment of the second one which illustrates a formal (3+2)‐cycloaddition. In consequence, this interaction leads to the C−C bond formation between C^3^ and C^2^ atoms of both molecules (Figure 2 B, step III). This bond formation is accompanied by the transfer of the respective unpaired electron to C^3^ of the second thiochalcone molecule. Finally, intramolecular recombination of both radical centers (Figure 2 B, step IV) leads to a driving force of −105 kJ mol^−1^ and the selective formation of the cycloadduct **3 a**.

The reaction of **1 a** with Fe_3_(CO)_12_ was repeated in a 1:1 ratio to increase the reduction equivalents and subsequently the amount of **3 a**. In this experiment the yield of **3 a** was esta‐ blished to 32 % and of **2 a** to 7 %, respectively.

The same ratio (1:1) was applied for reactions of thiochalcones **1 b**–**e** with Fe_3_(CO)_12_. The resulting products **2 b**–**e** (Figures S5–S8) and **3 b**–**e** were also formed side by side, but their ratios depended on Ar^1^ and Ar^2^ (Scheme 2, bottom). Similar to **3 a**, formation of **3 b**–**e** occurred with complete regio‐ and diastereoselectivity, evident by ^1^H and ^13^C{^1^H} NMR spectra registered for the isolated fractions. In all cases only one set of signals was observed. In analogy to **3 a**, complexes **3 d**,**e** (Figures S9, S10) feature the relative configuration of R*_C1_ R*_C2_ S*_C3_ S*_C6_. Again, complexes **3** can be considered as mimics of the H‐cluster of the naturally occurring [FeFe] hydrogenases,^16^ thus they are also of potential interest for the development of catalytic hydrogen production processes. Therefore, their redox properties were evaluated in the absence and presence of a proton source, that is, acetic acid (AcOH), using cyclic voltammetry (CV). These CV experiments showed, that **3 a**–**e** catalyze the reduction of protons in the presence of AcOH (Figures S12, S13).

The reaction of **1 b** with Fe_3_(CO)_12_ deserves a word of comment. In this case, formation of the dinuclear complex **4** along with **2 b** and **3 b**, was also observed (Scheme 2). After isolation, the identity of **4** was proven by spectroscopic methods and X‐ray analysis (Figure S11). The structure of **4** resembles the product obtained after reaction of 7,7‐dimethyl‐6,8‐dioxa‐2‐selenaspiro[3.4]nonane with Fe_3_(CO)_12_.^17^ Most likely, complex **4** was formed via reaction of the corresponding dimer B with Fe_3_(CO)_12_.

It is worth mentioning that blue colored dimers of type **B** are relatively stable in solution. In extension of the study, dimer B derived from **1 c** was isolated and characterized by X‐ray crystallography (Figure S4).

In summary, the presented study showed that “capricious” monomeric and dimeric thiochalcones react with Fe_3_(CO)_12_ yielding products of type **2**, **3** and **4**. In particular, diastereoselective formation of the five‐membered ring in complexes **3** containing four stereogenic centers is an intriguing finding. A plausible reaction mechanism involves an unprecedented, stepwise (3+2)‐cycloaddition of two thiochalcone molecules bonded to Fe_3_(CO)_12_ via the sulfur atoms. Quantum chemical calculations suggest that this reaction proceeds via a sequential intramolecular radical transfer initiated by the homolytic cleavage of one carbon‐sulfur bond resulting in formation of a reactive diradical intermediate. Thus, the described results demonstrate once more the unique ability of the sulfur atom incorporated into the C=S bond to promote diradical mechanisms governing the stepwise cycloaddition reactions of thiocarbonyl compounds.^18^

## Conflict of interest

The authors declare no conflict of interest.

## Supporting information

As a service to our authors and readers, this journal provides supporting information supplied by the authors. Such materials are peer reviewed and may be re‐organized for online delivery, but are not copy‐edited or typeset. Technical support issues arising from supporting information (other than missing files) should be addressed to the authors.

Supplementary

## References

[1a] G. Mloston , P. Grzelak , R. Hamera-Faldyga , M. Jasinski , P. Pipiak , K. Urbaniak , L. Albrecht , J. Hejmanowska , H. Heimgartner , Phosphorus Sulfur Silicon Relat. Elem. 2017, 192, 204;

[1b] P. Matczak , G. Mloston , R. Hamera-Faldyga , H. Görls , W. Weigand , Molecules 2019, 24, 3950. 10.3390/molecules24213950 PMC6864675 31683693

[2a] R. Huisgen , L. Fisera , H. Giera , R. Sustmann , J. Am. Chem. Soc. 1995, 117, 9671;

[2b] R. Huisgen , E. Langhals , Heteroat. Chem. 2006, 17, 433;

[2c] R. Huisgen , X. Li , H. Giera , E. Langhals , Helv. Chim. Acta 2001, 84, 981.

[3a] J. Breu , P. Höcht , U. Rohr , J. Schatz , J. Sauer , Eur. J. Org. Chem. 1998, 2861;

[3b] U. Rohr , J. Schatz , J. Sauer , Eur. J. Org. Chem. 1998, 2875.

[4a] G. M. Li , S. Niu , M. Segi , K. Tanaka , T. Nakajima , R. A. Zingaro , J. H. Reibenspies , M. B. Hall , J. Org. Chem. 2000, 65, 6601; 11052108 10.1021/jo000740q

[4b] G. Mloston , P. Grzelak , H. Heimgartner , J. Sulfur Chem. 2017, 38, 1;

[4c] G. Mloston , R. Hamera-Faldyga , H. Heimgartner , J. Sulfur Chem. 2018, 39, 322. 10.1039/c8ob01022f 29850725

[5a] G. Mloston , K. Urbaniak , R. Zimmer , H.-U. Reissig , H. Heimgartner , ChemistrySelect 2018, 3, 11724;

[5b] G. Mloston , K. Urbaniak , M. Jasinski , E.-U. Würthwein , H. Heimgartner , R. Zimmer , H.-U. Reissig , Chem. Eur. J. 2020, 26, 237; 31429509 10.1002/chem.201903385 PMC6973135

[5c] J. Hejmanowska , M. Jasinski , J. Wojciechowski , G. Mloston , L. Albrecht , Chem. Commun. 2017, 53, 11472. 10.1039/c7cc06518c 28984320

[6] P. Grzelak , G. Utecht , M. Jasinski , G. Mloston , Synthesis 2017, 49, 2129.

[7a] J.-P. Pradere , G. Bouet , H. Quiniou , Tetrahedron Lett. 1972, 13, 3471;

[7b] P. Beslin , D. Lagain , J. Vialle , C. Minot , Tetrahedron 1981, 37, 3839;

[7c] T. Karakasa , S. Satsumabayashi , S. Motoki , Bull. Chem. Soc. Jpn. 1986, 59, 335;

[7d] S. Motoki , T. Saito , T. Karakasa , T. Matsushita , E. Furuno , J. Chem. Soc. Perkin Trans. 1 1992, 2943;

[7e] A. Capperucci , A. Degl′Innocenti , S. Biondi , T. Nocentini , G. Rinaudo , Tetrahedron Lett. 2003, 44, 2831.

[8a] H. Alper , A. S. K. Chan , J. Chem. Soc. D 1971, 1203;

[8b] A. Q. Daraosheh , H. Görls , M. El-khateeb , G. Mloston , W. Weigand , Eur. J. Inorg. Chem. 2011, 349;

[8c] A. Q. Daraosheh , U.-P. Apfel , H. Görls , C. Friebe , U. S. Schubert , M. El-khateeb , G. Mloston , W. Weigand , Eur. J. Inorg. Chem. 2012, 318;

[8d] A. Q. Daraosheh , H. Abul-Futouh , H. Görls , W. Weigand , Inorg. Chim. Acta 2020, 503, 119377.

[9a] I. P. Georgakaki , L. M. Thomson , E. J. Lyon , M. B. Hall , M. Y. Darensbourg , Coord. Chem. Rev. 2003, 238, 255;

[9b] C. Tard , C. J. Pickett , Chem. Rev. 2009, 109, 2245; 19438209 10.1021/cr800542q

[9c] W. Lubitz , H. Ogata , O. Rüdiger , E. Reijerse , Chem. Rev. 2014, 114, 4081; 24655035 10.1021/cr4005814

[9d] U.-P. Apfel , F. Y. Petillon , P. Schollhammer , J. Talarmin , W. Weigand , Bioinspired Catalysis: Metal-Sulfur Complexes, Wiley-VCH, Weinheim, 2015, p. 97;

[9e] Y. Li , T. B. Rauchfuss , Chem. Rev. 2016, 116, 7043. 27258046 10.1021/acs.chemrev.5b00669 PMC4933964

[10a] W. Imhof , A. Göbel , D. Braga , P. De Leonardis , E. Tedesco , Organometallics 1999, 18, 736;

[10b] H.-J. Knölker , Chem. Soc. Rev. 1999, 28, 151;

[10c] H.-J. Knölker , Chem. Rev. 2000, 100, 2941; 11749311 10.1021/cr990267k

[10d] G. R. Stephenson , Science of Synthesis Vol. 1, Thieme Chemistry, 2014, p. 87.

[11] H. Alper , D. A. Brandes , Organometallics 1991, 10, 2457.

[12a] P. R. Nagy , M. Kállay , J. Chem. Theory Comput. 2019, 15, 5275; 31465219 10.1021/acs.jctc.9b00511

[12b] P. R. Nagy J. Chem. Theory Comput. 2018, 14, 4193. 29965753 10.1021/acs.jctc.8b00442

[13a] G. Henkelman , B. P. Uberuaga , H. Jonsson , J. Chem. Phys. 2000, 113, 9901;

[13b] G. Henkelman , H. Jonsson , J. Chem. Phys. 2000, 113, 9978.

[14] V. N. Staroverov , G. E. Scuseria , J. Tao , J. P. Perdew , J. Chem. Phys. 2003, 119, 12129.

[15] F. Weigend , R. Ahlrichs , Phys. Chem. Chem. Phys. 2006, 8, 1057.16633586

[16a] J. W. Peters , W. N. Lanzilotta , B. J. Lemon , L. C. Seefeldt , Science 1998, 282, 1853; 9836629 10.1126/science.282.5395.1853

[16b] Y. Nicolet , C. Piras , P. Legrand , C. E. Hatchikian , J. C. Fontecilla-Camps , Structure 1999, 7, 13. 10368269 10.1016/s0969-2126(99)80005-7

[17] R. Trautwein , H. Abul-Futouh , H. Görls , W. Imhof , L. R. Almazahreh , W. Weigand , New J. Chem. 2019, 43, 12580.

[18a] R. Huisgen , G. Mloston , K. Polborn , R. Sustmann , Chem. Eur. J. 2003, 9, 2256; 12772300 10.1002/chem.200204659

[18b] G. Mloston , K. Urbaniak , A. Linden , H. Heimgartner , Helv. Chim. Acta 2015, 98, 453;

[18c] G. Mloston , P. Pipiak , H. Heimgartner , Beilstein J. Org. Chem. 2016, 12, 716; 27340463 10.3762/bjoc.12.71 PMC4901872

[18d] G. Mloston , P. Grzelak , A. Linden , H. Heimgartner , Chem. Heterocycl. Compd. 2017, 53, 518.

