# Supplementary material for: Iron(0)‐Mediated Stereoselective (3+2)‐Cycloaddition of Thiochalcones via a Diradical Intermediate
Source: Chemistry. 2020 Aug 10;26(50):11412–6. doi: 10.1002/chem.202001412 (PMC7540601; doi:10.1002/chem.202001412)
Supplement: Supplementary file 1 — Supplementary [file CHEM-26-11412-s001.pdf]

Chemistry–A European Journal

Supporting Information

Iron(0)-Mediated Stereoselective (3+2)-Cycloaddition of Thiochalcones via a Diradical Intermediate

Philipp Buday+,[a] Phillip Seeber+,[b] Clara Zens,[b] Hassan Abul-Futouh,[c] Helmar Görls,[a] Stefanie Gräfe,[b] Piotr Matczak,[d] Stephan Kupfer,*[b] Wolfgang Weigand,*[a] and Grzegorz Mloston*[d]

Table of contents

1 Experimental procedures

1.1 General information

1.2 Synthesis and isolation of the iron complexes

1.3 Characterization of the iron complexes

1.4 Computational details

1.5 Structure determinations

2 Molecular structures

3 Cyclic voltammetry

4 NMR spectra

5 References

1 Experimental procedures

1.1 General information

Thiochalcones **1a**-**e** were synthesized by treatment of corresponding chalcones with Lawesson's reagent in accordance with established literature procedures starting from the corresponding ketones and aldehydes,[1] triiron dodecarbonyl was prepared from iron pentacarbonyl following a known protocol.[2] Syntheses of complexes **2a**-**e**, **3a**-**e** and **4** were performed under N_2 atmosphere by using standard Schlenk technique. Tetrahydrofuran used in all reactions was dried and deoxygenated over Na metal. Solvents used for column chromatography were distilled before the usage. All other solvents as well as commercially available compounds were purchased (Sigma-Aldrich, Acros, TCI, Alfa Aesar, abcr) and used as received without further purification. Geduran Si 60 (0.063-0.200 mm) was used as the stationary phase for column chromatography; TLC monitoring was performed on TLC Silica gel 60 F254.

Decomposition points were determined with APOTEC apparatus and are uncorrected. NMR spectra were recorded with Bruker 600 MHz and 400 MHz spectrometers (Avance III) using dried and deoxygenated $CDCl_3$ as solvent. IR spectra were measured with a Tensor 27 FT-IR spectrometer. DIP-EI mass spectrometry (70 eV) was performed with a Finnigan MAT SSQ 710. Elemental analysis was performed using an Euro Vector EA3000 element analyser. For single crystal X-Ray diffraction a Nonius KappaCCD diffractometer was used.

Cyclic voltammetry for complexes **3a**-**e** was accomplished with a three-electrode setup (working electrode: glassy carbon (d = 1.6 mm); reference electrode: Ag/Ag^+ in acetonitrile; counter electrode: Pt (wire)) using a Reference 600 Potentiostat/Galvanostat/ZRA from Gamry Instruments. The measurements were conducted in deoxygenated CH_2Cl_2-[n-Bu_4N][BF_4] (0.1 M) solutions with a complex concentration of 1 mM. After each measurement the glassy carbon electrode was polished with alumina. To ensure oxygen-free conditions, solution and headspace were purged with N_2 while opening the system, during the runs only the headspace was purged. The cyclic voltammograms were referenced against the ferrocenium/ferrocene (Fc^+/Fc) couple.

1.2 Synthesis and isolation of the iron complexes

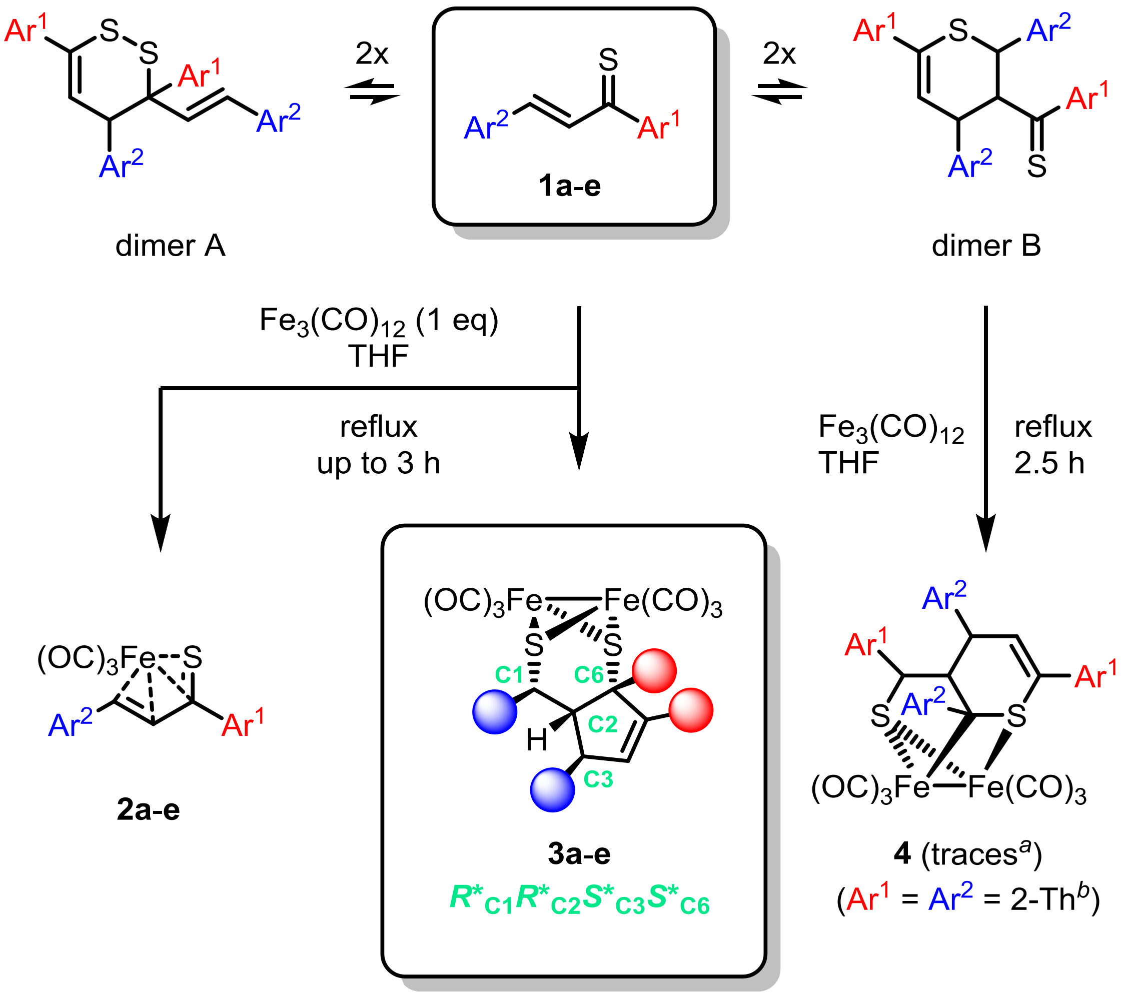

	Ar[1] / (red)	Ar[2] / (blue)	isolated yield[d] **2**	**3**
**a**	2-Th[b]	Ph	7 %	32 %
**b**	2-Th[b]	2-Th[b]	20 %	15 %
**c**	Ph	Ph	21 %	31 %
**d**	Fc[c]	Ph	33 %	17 %
**e**	Fc[c]	2-Th[b]	24 %	14 %

Scheme S1. Thiochalcones **1a-e** reactions with $Fe_3(CO)_{12}$ resulting in mono and cyclic dinuclear iron complexes **2a-e** and **3a-e**, respectively, as well as in thiopyran complex **4** (isolated for substituents $Ar^1 = Ar^2$ = 2-Th[b], only). [a]Unexpectedly, a second experiment with another sample of **1b** resulted in a better yield of **4.** [b]2-Th = thien-2-yl. [c]Fc = ferrocenyl. [d]Calculated for the reactions of **1a-e** with $Fe_3(CO)_{12}$ in 1:1 ratio (in the case of 3:1 ratio **2a** was obtained in 22 % yield and **3a** in 6 % yield).

General procedure for the synthesis and purification of mono (**2a**-**e**) (Scheme S1) and cyclic dinuclear iron complexes (**3a**-**e**), exemplarily shown for **2a** and **3a**:

1-(Thien-2-yl)-3-phenylprop-2-enethione **1a** (51 mg, 0.22 mmol, 1 eq) and $Fe_3(CO)_{12}$ (110 mg, 0.22 mmol, 1 eq) were placed in a three-necked Schlenk flask equipped with a condenser and dissolved in a portion of THF (15 mL). The green solution was slowly heated to reflux for 1.5 h (until no more $Fe_3(CO)_{12}$ was observed by TLC). Next, the solvent was removed in a nitrogen flow and the dark brown residue was purified by column chromatography (*n*-hexane:CH_2Cl_2 = 3:1) affording **2a** (5.7 mg, 0.015 mmol, 7 %) and **3a** (25.7 mg, 0.035 mmol, 32 %) as red solids. Single crystals suitable for X-Ray diffraction were obtained by slow evaporation of concentrated *n*-hexane solutions of **2a** and **3a**.

Procedure for the synthesis and isolation of thiopyran complex **4**:

The general procedure as shown above using **1b** as thiochalcone yielded **4** only in traces. According to the same procedure, a second experiment performed with another sample of **1b** resulted in an enhanced yield of **4** (26 %). This result can be explained by the presence of a variable ratio of dimers A and B (Scheme S1) as major components of the “thiochalcone fractions” obtained after column chromatography.

1.3 Characterization of the iron complexes

General comment: NMR signal assignment for complexes **2** is based on reported literature.[3]

Compound **2a**

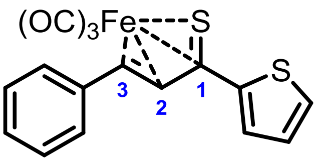

Yield: 6 mg (7 %) of red solid after drying in vacuo;

R_f = 0.49 (*n*-hexane:CH_2Cl_2 3:1);

Decomposition point: > 107 °C (slow decomposition);

^{1}H NMR (400 MHz, $CDCl_3$): δ = 7.75 (dd, $J_{H,H}$ = 3.80 Hz, 1H, Ar-*H*), 7.24-7.45 (m, 6H, Ar-*H* and $CDCl_3$ residual signal), 7.05-7.10 (m, 1H, Ar-*H*), 6.91 (d, $^3J_{H,H}$ = 9.35 Hz, 1H, *H*2), 3.34 ppm (d, $^3J_{H,H}$ = 9.65 Hz, 1H, *H*3);

^{13}C NMR (101 MHz, $CDCl_3$): δ = 71.1 (*C*3), 85.9 (*C*2), 109.6, 125.9, 126.7, 127.0, 127.7, 127.9, 129.1, 137.8, 145.7 ppm;

IR: $\tilde{\nu}$ = 1971 (vs), 1992 (s), 2003 (s), 2049 (vs) cm^{-1} (CO);

MS (EI, 70 eV): *m*/*z* (%) = 370 (10) [*M*]$^+$, 342 (20) [*M*-CO]$^+$, 314 (24) [*M*-2 CO]$^+$, 286 (100) [*M*-3 CO]$^+$;

EA: calcd (%) for $C_{16}H_{10}FeO_3S_2$: C 51.91, H 2.72, S 17.32; found C 52.29, H 2.71, S 17.59.

Compound **2b**

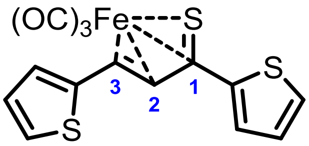

Yield: 33 mg (20 %) of red solid after drying in vacuo;

R_f = 0.49 (*n*-hexane:CH_2Cl_2 3:1);

Decomposition point: > 108 °C (slow decomposition);

^{1}H NMR (400 MHz, $CDCl_3$): δ = 7.72 (dd, $J_{H,H}$ = 3.51, 0.88 Hz, 1H, Ar-*H*), 7.37 (dd, $J_{H,H}$ = 5.26, 0.88 Hz, 1H, Ar-*H*), 7.24 (d, $J_{H,H}$ = 4.97 Hz, 1H, Ar-*H*), 7.15 (d, $J_{H,H}$ = 3.15 Hz, 1H, Ar-*H*), 7.07 (dd, $J_{H,H}$ = 4.97, 3.80 Hz, 1H, Ar-*H*), 6.97 (dd, $J_{H,H}$ = 4.97, 3.80 Hz, 1H, Ar-*H*), 6.74 (d, $^3J_{H,H}$ = 9.35 Hz, 1H, *H*2), 3.63 ppm (d, $^3J_{H,H}$ = 9.06 Hz, 1H, *H*3);

^{13}C NMR (151 MHz, $CDCl_3$): δ = 66.4 (*C*3), 88.3 (*C*2), 108.7, 124.9, 125.7, 125.9, 127.1, 128.0, 128.1, 143.0, 145.3 ppm;

IR: $\tilde{v}$ = 1981 (vs), 2055 (s) cm^{-1} (CO);

MS (EI, 70 eV): *m*/*z* (%) = 376 (8) [*M*]$^+$, 348 (20) [*M*-CO]$^+$, 320 (36) [*M*-2 CO]$^+$, 292 (100) [*M*-3 CO]$^+$;

EA: calcd (%) for $C_{14}H_8FeO_3S_3$: C 44.69, H 2.14, S 25.56; found C 44.40, H 2.11, S 25.34.

Compound **2c**

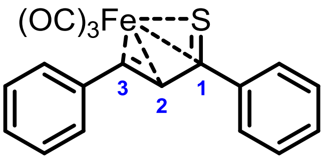

Yield: 38 mg (21 %) of red solid after drying in vacuo;

R_f = 0.49 (*n*-hexane:CH_2Cl_2 3:1);

Decomposition point: > 110 °C (slow decomposition);

^{1}H NMR (600 MHz, $CDCl_3$): δ = 7.98-8.04 (m, 2H, Ar-*H*), 7.39-7.49 (m, 5H, Ar-*H*), 7.31-7.37 (m, 2H, Ar-*H*), 7.27-7.30 (m, 1H, Ar-*H*), 6.94 (d, $^3J_{H,H}$ = 9.35 Hz, 1H, *H*2), 3.34 ppm (d, $^3J_{H,H}$ = 9.54 Hz, 1H, *H*3);

^{13}C NMR (151 MHz, $CDCl_3$): δ = 72.3 (*C*3), 87.6 (*C*2), 115.8, 126.8, 127.5, 127.7, 128.7, 129.1, 129.7, 138.0, 139.6 ppm;

IR: $\tilde{v}$ = 1935 (vs), 1956 (vs), 1963 (vs), 1982 (s), 1996 (vs), 2012 (s), 2054 (vs) cm^{-1} (CO);

MS (EI, 70 eV): *m*/*z* (%) = 364 (8) [*M*]$^+$, 336 (12) [*M*-CO]$^+$, 308 (20) [*M*-2 CO]$^+$, 280 (100) [*M*-3 CO]$^+$;

EA: calcd (%) for $C_{18}H_{12}FeO_3S$: C 59.36, H 3.32, S 8.80; found C 59.52, H 3.23, S 8.89.

Compound **2d**

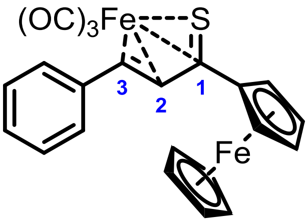

Yield: 48 mg (33 %) of red solid after drying in vacuo;

R_f = 0.38 (*n*-hexane:CH_2Cl_2 3:1);

Decomposition point: > 125 °C (slow decomposition);

^{1}H NMR (400 MHz, $CDCl_3$): δ = 7.37-7.42 (m, 2H, Ar-*H*), 7.30-7.36 (m, 2H, Ar-*H*), 7.23-7.27 (m, 1H, Ar-*H*), 6.56 (d, $^3J_{H,H}$ = 9.35 Hz, 1H, *H*2), 4.91-4.98 (m, 2H, Cp-*H*), 4.44-4.52 (m, 2H, Cp-*H*), 4.21 (s, 5H, Cp-*H*), 3.37 ppm (d, $^3J_{H,H}$ = 9.35 Hz, 1H, *H*3);

^{13}C NMR (400 MHz, $CDCl_3$): δ = 65.6, 69.2, 70.2, 70.3, 70.4, 70.4, 84.7, 85.2, 118.1 (*C*2), 126.7, 127.4, 129.0, 138.4 ppm.

IR: $\tilde{\nu}$ = 1970 (s), 1984 (m), 2005 (s), 2043 (m), 2052 (s) cm^{-1} (CO);

MS (EI, 70 eV): *m*/*z* (%) = 472 (8) [*M*]$^+$, 416 (24) [*M*-2 CO]$^+$, 388 (100) [*M*-3 CO]$^+$;

EA: calcd (%) for $C_{22}H_{16}Fe_2O_3S$: C C 55.97, H 3.42, S 6.79; found C 56.35, H 3.36, S 6.41.

Compound **2e**

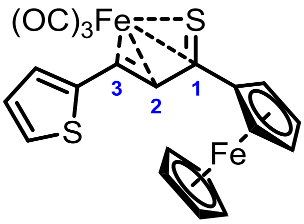

Yield: 34 mg (24 %) of red solid after drying in vacuo;

R_f = 0.38 (*n*-hexane:CH_2Cl_2 3:1);

Decomposition point: > 135 °C (slow decomposition);

^{1}H NMR (400 MHz, $CDCl_3$): δ = 7.21 (d, $J_{H,H}$ = 5.26 Hz, 1H, Ar-*H*), 7.12 (d, $J_{H,H}$ = 3.51 Hz, 1H, Ar-*H*), 6.96 (dd, $J_{H,H}$ = 4.97, 3.80 Hz, 1H, Ar-*H*), 6.39 (d, $J_{H,H}$ = 9.06 Hz, 1H, *H*2), 4.88-4.95 (m, 2H, Cp-*H*), 4.47 (dd, $J_{H,H}$ = 12.13, 1.32 Hz, 2H, Cp-*H*), 4.21 (s, 5H, Cp-*H*), 3.66 ppm (d, $^3J_{H,H}$ = 9.35 Hz, 1H, *H*3);

^{13}C NMR (151 MHz, $CDCl_3$): δ = 65.6, 65.6 (*C*3, Cp-*C*), 69.2, 70.2, 70.4, 70.4 (Cp-*C*), 84.8, 85.2 (*C*2), 117.3, 124.5, 125.3, 128.0, 143.7 ppm.

IR: $\tilde{\nu}$ = 1976 (vs), 2049 (s) cm^{-1} (CO);

MS (EI, 70 eV): *m*/*z* (%) = 478 (24) [*M*]$^+$, 422 (28) [*M*-2 CO]$^+$, 394 (100) [*M*-3 CO]$^+$;

EA: calcd (%) for $C_{20}H_{14}Fe_2O_3S_2$: C 50.24, H 2.95, S 13.41; found C 50.58, H 3.04, S 13.49.

Compound **3a**

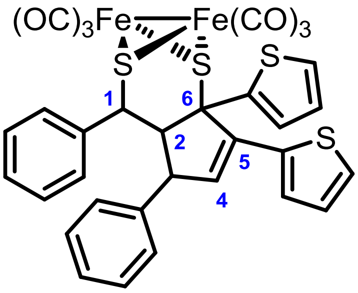

Yield: 26 mg (32 %) of red solid after drying in vacuo;

R_f = 0.33 (*n*-hexane:CH_2Cl_2 3:1);

Decomposition point: > 160 °C (slow decomposition);

^{1}H NMR ($CDCl_3$, 400 MHz): δ = 7.38-7.45 (m, 2H, Ar-*H*), 7.09-7.20 (m, 4H, Ar-*H*), 6.86-7.02 (m, 8H, Ar-*H*), 6.78 (dd, $J_{H,H}$ = 7.60, 1.46 Hz, 2H, Ar-*H*), 5.98 (d, $J_{H,H}$ = 2.05 Hz, 1H, *H*4), 4.08 (dd, $J_{H,H}$ = 8.18, 2.05 Hz, 1H, *H*3), 3.85 (d, $J_{H,H}$ = 4.38 Hz, 1H, *H*1), 3.56 ppm (dd, $J_{H,H}$ = 8.18, 4.38 Hz, 1H, *H*2);

^{13}C NMR ($CDCl_3$, 101 MHz): δ = 43.6 (*C*1), 51.0 (*C*3), 59.2 (*C*6), 65.4 (*C*2), 125.5, 125.9, 126.0, 126.6, 126.9, 127.0, 127.3, 127.7, 127.8, 127.9, 128.0, 128.7, 136.1 (*C*4), 136.2, 140.2, 142.0, 142.5, 148.3, 207.3 ppm (*C*O);

IR: $\tilde{\nu}$ = 1967 (vs), 2027 (vs), 2070 (vs) cm^{-1} (CO);

MS (EI, 70 eV): *m*/*z* (%) = 684 (1) [*M*-2 CO]$^+$, 656 (6) [*M*-3 CO]$^+$, 628 (2) [*M*-4 CO]$^+$, 600 (5) [*M*-5 CO]$^+$, 572 (36) [*M*-6 CO]$^+$;

EA: calcd (%) for $C_{32}H_{20}Fe_2O_6S_4$: C 51.91, H 2.72, S 17.32; found C 51.87, H 2.64, S 17.37.

Compound **3b**

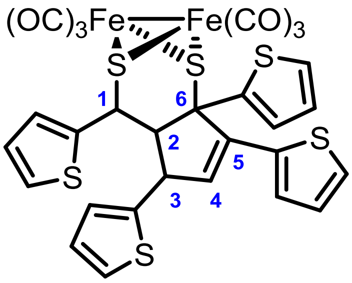

Yield: 24 mg (15%) of red solid after drying in vacuo;

R_f = 0.30 (*n*-hexane:CH_2Cl_2 3:1);

Decomposition point: > 160 °C (slow decomposition);

^{1}H NMR ($CDCl_3$, 600 MHz): δ = 7.39 (dd, $J_{H,H}$ = 5.14, 1.10 Hz, 1H, Ar-*H*), 7.34 (dd, $J_{H,H}$ = 3.58, 1.19 Hz, 1H, Ar-*H*), 7.19 (dd, $J_{H,H}$ = 5.04, 1.01 Hz, 1H, Ar-*H*), 7.09 (dd, $J_{H,H}$ = 5.14, 3.67 Hz, 1H, Ar-*H*), 7.01 (dd, $J_{H,H}$ = 5.14, 1.10 Hz, 1H, Ar-*H*), 6.98 (d, $J_{H,H}$ = 4.77 Hz, 1H, Ar-*H*), 6.91 (dd, $J_{H,H}$ = 5.04, 3.76 Hz, 1H, Ar-*H*), 6.83-6.87 (m, 2H, Ar-*H*), 6.70 (dd, $J_{H,H}$ = 5.05, 3.58 Hz, 1H, Ar-*H*), 6.61 (dd, $J_{H,H}$ = 5.14, 3.48 Hz, 1H, Ar-*H*), 6.28 (dd, $J_{H,H}$ = 3.48, 0.73 Hz, 1H, Ar-*H*), 6.07 (d, $J_{H,H}$ = 2.20 Hz, 1H, *H*4), 4.36

(dd, $J_{H,H}$ = 7.98, 2.11 Hz, 1H, *H*3), 4.04 (d, $J_{H,H}$ = 4.58 Hz, 1H, *H*1), 3.58 ppm (dd, $J_{H,H}$ = 7.98, 4.68 Hz, 1H, *H*2);

^{13}C NMR ($CDCl_3$, 101 MHz): δ = 39.2 (*C*1), 46.4 (*C*3), 58.7 (*C*6), 67.7 (*C*2), 123.6, 124.4, 125.0, 125.8, 125.8, 126.3, 126.3, 126.6, 126.8, 127.0 (2 C atoms), 128.4, 134.9, 135.9, 142.3, 144.5, 147.4, 148.1, 207.0 ppm (*C*O);

IR: $\tilde{\nu}$ = 1969 (vs), 2028 (vs), 2072 (vs) cm^{-1} (CO);

MS (EI, 70 eV): *m*/*z* (%) = 752 (<1) [*M*]$^+$, 696 (2) [*M*-2 CO]$^+$, 668 (8) [*M*-3 CO]$^+$, 640 (1) [*M*-4 CO]$^+$, 612 (8) [*M*-5 CO]$^+$, 584 (100) [*M*-6 CO]$^+$;

EA: calcd (%) for $C_{28}H_{16}Fe_2O_6S_6$: C 44.69, H 2.14, S 25.56; found C 45.76, H 2.38, S 25.75.

Compound **3c**

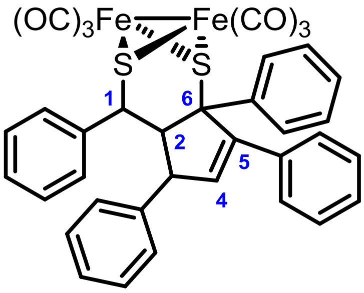

Yield: 56 mg (31 %) of red solid after drying in vacuo;

R_f = 0.34 (*n*-hexane:CH_2Cl_2 3:1);

Decomposition point: 180-185 °C (rapid decomposition);

^{1}H NMR ($CDCl_3$, 600 MHz): δ = 7.82 (d, $J_{H,H}$ = 7.70 Hz, 2H, Ar-*H*), 7.43 (t, $J_{H,H}$ = 7.70 Hz, 2H, Ar-*H*), 7.32-7.37 (m, 1H, Ar-*H*), 7.23-7.27 (m, 2H, Ar-*H*), 7.13-7.21 (m, 3H, Ar-*H*), 7.07 (d, $J_{H,H}$ = 7.52 Hz, 2H, Ar-*H*), 6.87-6.99 (m, 6H, Ar-*H*), 6.84 (d, $J_{H,H}$ = 7.15 Hz, 2H, Ar-*H*), 6.17 (d, $J_{H,H}$ = 2.02 Hz, 1H, *H*4), 4.23 (dd, $J_{H,H}$ = 8.25, 1.83 Hz, 1H, *H*3), 3.72 (d, $J_{H,H}$ = 4.03 Hz, 1H, *H*1), 3.42 ppm (dd, $J_{H,H}$ = 8.25, 4.22 Hz, 1H, *H*2);

^{13}C NMR (151 MHz, $CDCl_3$): δ = 44.3 (*C*1), 51.2 (*C*3), 62.5 (*C*6), 63.9 (*C*2), 125.9, 127.2, 127.5, 127.6, 127.7, 127.9, 127.9, 127.9, 127.9, 128.1, 128.4, 128.5, 134.0 (Ar-*C*), 137.3 (*C*4), 140.4, 142.8, 144.1, 147.1 (Ar-*C*), 207.5 ppm (*C*O);

IR: $\tilde{\nu}$ = 1974 (vs), 1981 (vs), 1994 (vs), 2013 (s), 2033 (s), 2073 (s) cm^{-1} (CO);

MS (EI, 70 eV): *m*/*z* (%) = 728 (1) [*M*]$^+$, 672 (2) [*M*-2 CO]$^+$, 644 (8) [*M*-3 CO]$^+$, 616 (12) [*M*-4 CO]$^+$, 588 (14) [*M*-5 CO]$^+$, 560 (100) [*M*-6 CO]$^+$;

EA: calcd (%) for $C_{36}H_{24}Fe_2O_6S_2$: C 59.36, H 3.32, S 8.80; found C 59.44, H 3.34, S 8.55.

Compound **3d**

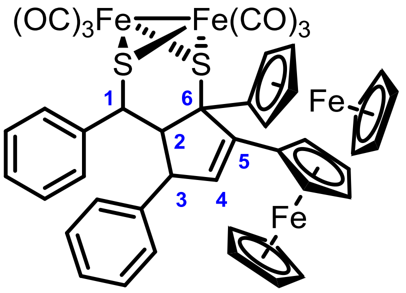

Yield: 24 mg (17 %) of red solid after drying in vacuo;

R_f = 0.28 (*n*-hexane:CH_2Cl_2 3:1);

Decomposition point: 185 °C (rapid decomposition);

^{1}H NMR ($CDCl_3$, 400 MHz): δ = 7.25 (m, 1H), 6.87-7.12 (m, 6H), 6.78 (d, $J_{H,H}$ = 5.26 Hz, 2H), 6.09 (m, 1H, *H*4), 5.38 (m, 1H), 4.46 (m, 1H), 4.14-4.41 (m, 12H), 4.06-4.12 (m, 1H), 4.01-4.05 (m, 1H), 3.98 (s, 5H, Cp-*H*), 3.50-3.61 ppm (m, 1H, *H*2);

^{13}C NMR ($CDCl_3$, 101 MHz): δ = 44.0 (*C*1), 50.5 (*C*3), 57.0 (*C*6), 59.1 (*C*2), 65,7, 67.0, 67.2, 67.9, 68.8, 69.2, 69.7, 73.9, 82.7, 92.7, 125.6, 127.3, 127.4, 127.9, 127.9, 128.1, 136.2, 141.1, 143.1, 146.2, 208.1 ppm (*C*O);

IR: $\tilde{\nu}$ = 1973 (vs), 1980 (s), 1986 (s), 1998 (s), 2024 (m), 2067 (m) cm^{-1} (CO);

MS (EI, 70 eV): *m*/*z* (%) = 944 (<1) $[M]^+$, 776 (1) $[M\text{-}6\ CO]^+$, 510 (100) $[C_{31}H_{26}Fe_2]^+$;

EA: calcd (%) for $C_{44}H_{32}Fe_4O_6S_2$*1.2C_6H_{14}: C 58.70, H 4.70, S 6.12; found C 58.86, H 4.46, S 6.05.

Compound **3e**

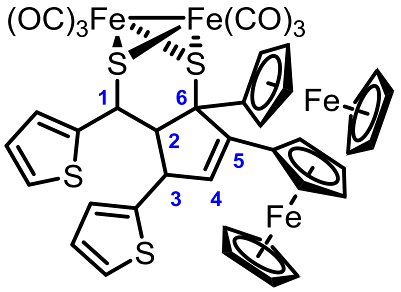

Yield: 20 mg (14 %) of red solid after drying in vacuo;

R_f = 0.30 (*n*-hexane:CH_2Cl_2 3:1);

Decomposition point: > 180 °C (slow decomposition);

^{1}H NMR ($CDCl_3$, 400 MHz): δ = 6.99-7.05 (m, 3H, Ar-*H*), 6.75 (dd, $J_{H,H}$ = 4.97, 3.80 Hz, 1H, Ar-*H*), 6.65 (dd, $J_{H,H}$ = 4.97, 3.51 Hz, 1H, Ar-*H*), 6.28 (dd, $J_{H,H}$ = 3.51, 0.88 Hz, 1H, Ar-*H*), 6.17 (d, $^3J_{H,H}$ = 2.05 Hz, 1H, *H*4), 5.26-5.30 (m, 1H, Cp-*H*), 4.45-4.50 (m, 1H, Cp-*H*), 4.38-4.43 (m, 1H, *H*3), 4.34-4.38 (m, 1H, Cp-*H*), 4.30-4.33 (m, 1H, Cp-*H*), 4.25-4.30 (m, 7H, *H*1 and Cp-*H*), 4.19-4.21 (m, 2H, Cp-*H*), 4.03-4.06 (m, 5H, Cp-*H*), 4.01-4.03 (m, 1H, Cp-*H*), 3.55 ppm (dd, $^3J_{H,H}$ = 8.18, 4.97 Hz, 1H, *H*2);

^{13}C NMR ($CDCl_3$, 101 MHz): δ = 40.1 (*C*1), 46.3 (*C*3), 59.0 (*C*6), 60.6 (*C*2), 65.6, 67.1, 67.3, 68.0, 68.9, 69.4, 69.5, 69.6, 69.7, 73.8, 82.3, 92.5 (Cp-*C*), 123.3, 124.7, 124.8, 125.7, 126.4, 126.5, 135.1 (*C*4), 145.1, 147.0, 147.9, 207.8 ppm (*C*O);

IR: $\tilde{\nu}$ = 1974 (s), 1981 (m), 1995 (m), 2017 (m), 2028 (m), 2071 (m) cm^{-1} (CO);

MS (EI, 70 eV): *m*/*z* (%) = 612 (8) [$C_{34}H_{28}Fe_2S_2$]$^+$, 516 (100) [$C_{29}H_{24}Fe_2S$]$^+$;

EA: calcd (%) for $C_{40}H_{28}Fe_4O_6S_4$*0.8C_6H_{14}: C 52.49, H 3.85, S 12.51; found C 52.38, H 3.51, S 12.49.

Compound **4**

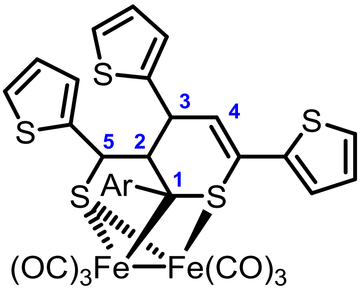

Ar = 2-Th

Yield: 126 mg (26 %, experiment with the better yield) of red solid after drying in vacuo;

R_f = 0.26 (*n*-hexane:CH_2Cl_2 3:1);

Decomposition point: > 150 °C (slow decomposition);

^{1}H NMR (600 MHz, $CDCl_3$): δ = 7.49 (d, $J_{H,H}$ = 4.95 Hz, 1H, Ar-*H*), 7.37 (d, $J_{H,H}$ = 4.95 Hz, 1H, Ar-*H*), 7.29 (d, $J_{H,H}$ = 3.12 Hz, 1H, Ar-*H*), 7.22-7.26 (m, 1H, Ar-*H*), 7.19 (d, $J_{H,H}$ = 2.93 Hz, 1H, Ar-*H*), 7.06 (dd, $J_{H,H}$ = 4.58, 3.85 Hz, 1H, Ar-*H*), 6.84 (d, $J_{H,H}$ = 4.95 Hz, 1H, Ar-*H*), 6.70 (d, $J_{H,H}$ = 4.95 Hz, 1H, Ar-*H*), 6.57 (d, $J_{H,H}$ = 2.38 Hz, 1H, Ar-*H*), 6.42-6.47 (m, 1H, Ar-*H*), 6.38-6.42 (m, 1H, Ar-*H*), 6.34 (d, $^3J_{H,H}$ = 5.50 Hz, 1H, *H*4), 6.06 (d, $^3J_{H,H}$ = 12.29 Hz, 1H), 5.99 (d, $J_{H,H}$ = 3.30 Hz, 1H, Ar-*H*), 4.01 (d, $^3J_{H,H}$ = 12.29 Hz, 1H), 3.56 ppm (d, $^3J_{H,H}$ = 5.87 Hz, 1H, *H*3);

^{13}C NMR (151 MHz, $CDCl_3$): δ = 37.9 (*C*3), 47.5 (*C*1), 52.6, 55.0, 119.7, 121.1, 123.3, 125.4, 125.5, 126.4, 126.5, 126.6, 126.9, 127.3, 128.2, 128.6, 129.5 (Ar-*C*), 130.0 (*C*4), 137.3, 143.7, 144.7, 157.4 (Ar-*C*), 207.3, 210.8, 211.2 (*C*O) ppm;

IR: $\tilde{\nu}$ = 1956 (vs), 1984 (vs), 2022 (vs), 2063 (vs) cm^{-1} (CO);

MS (EI, 70 eV): *m*/*z* (%) = 753 (2) [*M*]$^+$, 724 (8) [*M*-CO]$^+$, 696 (12) [*M*-2 CO]$^+$, 668 (66) [*M*-3 CO]$^+$, 640 (6) [*M*-4 CO]$^+$, 612 (20) [*M*-5 CO]$^+$, 584 (100) [*M*-6 CO]$^+$;

EA: calcd (%) for $C_{28}H_{16}Fe_2O_6S_6$: C 44.69, H 2.14, S 25.56; found C 44.39, H 2.16, S 25.30.

1.4 Computational details

In order to elucidate the reaction mechanism underlying the selective formation of the cycloadduct (**3a**), shown in Scheme 2 and Scheme S1, a suite of quantum chemical methods has been utilized. Initially, two rotamers of **1a** the *E* ($\mathbf{E_A}$ and $\mathbf{E_B}$) and the *Z* ($\mathbf{Z_A}$ and $\mathbf{Z_B}$) isomers of the free thiochalcones (Ar^1 = thiophenyl, Ar^2 = phenyl, see Figure S1) were fully optimized at the DF-HF+DF-MP2[4]/cc-pVTZ[5](cc-pVTZ/JKFIT+cc-pVTZ/C) level of theory using PSI4 1.2.1,[6] followed by a vibrational analysis to confirm that a minimum on the 3*N*-6 dimensional potential energy (hyper-)surface (PES) was obtained. Subsequently, the energy of $\mathbf{E_A}$, $\mathbf{E_B}$, $\mathbf{Z_A}$ and $\mathbf{Z_B}$ was recalculated within these optimized structures by LNO-CCSD(T)[7] and using the cc-pVQZ[5] basis set as implemented in the program MRCC.[8]

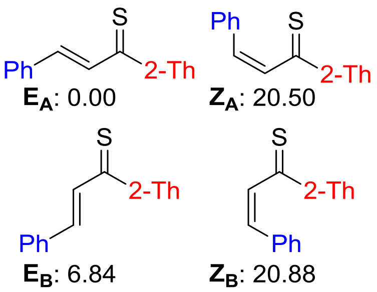

Figure S1. Relative Gibbs energies of **1a** (ΔG in kJ mol^{-1}), *i.e.*, two rotamers of *E* ($\mathbf{E_A}$ and $\mathbf{E_B}$) and *Z* ($\mathbf{Z_A}$ and $\mathbf{Z_B}$) isomers within the fully optimized equilibrium structures, obtained at the DF-MP2/cc-pVTZ level of theory. The electronic energy contribution was subsequently obtained at the LNO-CCSD(T)/cc-pVQZ level of theory.

In the following, two molecules of thiochalcone are coordinated to the $[Fe_2(CO)_6]$ fragment forming the respective adduct (**A**), while all combinations of $\mathbf{E_A}$, $\mathbf{E_B}$, $\mathbf{Z_A}$ and $\mathbf{Z_B}$ were considered, see Table S1 (left) exemplarily for $\mathbf{A(E_AE_A)}$. In addition to the ten possible combinations of the four isomers, coordination in a parallel as well as in an antiparallel fashion is conceivable. However, in the present study, we focused exclusively on adducts obtained by parallel coordination as the following reaction is highly unlikely to proceed starting from the antiparallel adducts, due to an unfavorable stereochemistry, *i.e.* regarding the required carbon-carbon bond formation (C2-C3') among the two thioketones. All ten (parallel coordinated) adducts were optimized using the TPSSh[9] hybrid functional and the def2-TZVPP[10] basis set as implemented in ORCA 4.1.1 and 4.2.1,[11] while the resolution of identity approach was applied, using the respective def2/J auxiliary basis and the RIJCOSX[12] approximation for the Coulomb and exchange integrals, to reduce the computational demand. Furthermore, D3 dispersion correction with Becke-Johnson damping (D3BJ)[13] was added to account for (attractive) long-range interactions among the coordinated thioketones. A subsequent frequency analysis confirmed the presence of minima on the PES. For the adduct $\mathbf{A(E_AE_A)}$, resulting from coordination of the most stable isolated thioketone ($\mathbf{E_A}$, Figure S1), the structural and electronic properties were additionally calculated within the equilibrated triplet ground state structure.

Table S1. Relative Gibbs energies of the adducts (ΔG in kJ mol^{-1}, **A**), formed upon coordination of the $[Fe_2(CO)_6]$ fragment by two molecules of **1a** ($\mathbf{E_A}$, $\mathbf{E_B}$, $\mathbf{Z_A}$ and $\mathbf{Z_B}$), respectively, and of the resulting products (**P**). All values are obtained for the closed-shell singlet ground state, triplet energies in

parenthesis, and given with respect to **A(E_AE_A)** and **P(E_AE_A)**, respectively. Selectively formed stereocenters (1, 2, 3 and 6) of the possible products are labeled; incorrectly formed centers are highlighted in red.

Adduct (**A**)	Product (**P**)
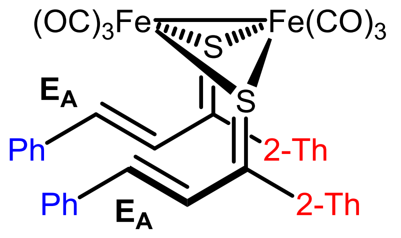	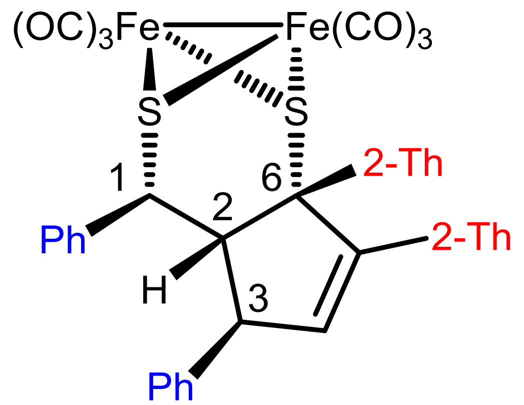

Adduct (**A**), ΔG / kJ mol^{-1}:

	E_A	E_B	Z_A	Z_B
E_A	0.00 (66.93)	-16.20	13.05	6.41
E_B	-	-5.96	7.31	18.24
Z_A	-	-	26.34	30.54
Z_B	-	-	-	35.86

Product (**P**), ΔG / kJ mol^{-1}:

	E_A	E_B	Z_A	Z_B
E_A	0.00 (52.53)<br>1236	41.49<br>1236	-	-4.42<br>1236
E_B	37.66<br>1236	-2.82<br>1236	40.37<br>1234	24.40<br>1234
Z_A	-	4.16<br>1236	-	0.86<br>1236
Z_B	69.42<br>1236	28.84<br>1236	30.54<br>1236	56.13<br>1236

Originating from these ten adducts, 16 products (**P**) are conceivable. These products are not symmetric – with one thioketone contributing three and the other thioketone contributing two carbon atoms to the formed cyclopentene moiety. Thus, the products are denoted accordingly: Firstly, the thioketone contributing three carbon atoms; secondly, the thioketone contributing two carbon atoms, *e.g.* **P(E_BZ_B)** *vs.* **P(Z_BE_B)**. The structural and electronic properties of these product structures, resulting from a formal (3+2) cycloaddition, were obtained at the same level of theory as described previously for the adducts. However, three of the constructed products, namely **P(E_AZ_A)**, **P(Z_AE_A)** and **P(Z_AZ_A)**, relax in already covered product states, thus a total number of 13 products is obtained. From these 13 products, merely two species – **P(E_AE_A)** and **P(Z_BE_A)** – feature the correct stereochemistry of all four chiral carbon atoms as observed experimentally, see Table S1 (right). These two products only differ with respect to the torsion of the thiophenyl (Ar^1) and the phenyl (Ar^2) groups. Furthermore, **P(E_AE_A)**, as obtained from the adduct **A(E_AE_A)** resulting from coordination of the most stable **1a** isomer (**E_A**, Figure S1), was optimized within triplet multiplicity, followed by a vibrational analysis. These computational results clearly show that both **A(E_AE_A)** and **P(E_AE_A)** are closed-shell singlet species.

In order to determine the reaction mechanism leading to the (3+2)-cycloproduct, the minimum energy path (MEP) along the reaction coordinate from the adduct to the respective product was approximated by the climbing image nudge elastic band (CI-NEB) method.[14] Based on the previously calculated thermodynamic properties of the free thiochalcones, their adducts and their respective products, the most plausible reaction coordinate connects **E_A**, via the adduct **A(E_AE_A)** to the final product **P(E_AE_A)**. Thus, the MEP, connecting **A(E_AE_A)** and **P(E_AE_A)**, was obtained at the TPSSh/def2-SVP[10] level of theory within singlet multiplicity (closed-shell); subsequently, open-shell singlet (images 6 to 9) and triplet single point calculations (TPSSh/def2-SVP) were obtained within all CI-NEB images (Figure S2 A).

In order to estimate the influence of solvent stabilization along the closed-shell singlet reaction coordinate, single point calculations (closed-shell singlet) including the tetrahydrofuran solvent by the SMD continuum model[15] were performed (Figure S2 B). The energy of all images is stabilized by an almost constant value of approximately 70 kJ mol^{-1}. However, an explicit description of solvent molecules might reduce the activation barrier further, as the homolytic carbon sulfur bond cleavage yields a highly reactive biradical intermediate. Furthermore, a triplet reaction path was optimized connecting the closed-shell adduct and product, **A(E$_A$E$_A$)** and **P(E$_A$E$_A$)**, respectively, see Figure S2 C. In addition, open-shell singlet energies were calculated along the triplet pathway. However, the resulting activation energy along the triplet coordinate is with roughly 310 kJ mol^{-1} (for both: open-shell singlet as well as triplet wavefunction) – more than 100 kJ mol^{-1} higher than the most favorable open-shell singlet solution along the singlet pathway (recall Figure 2 A and Figure S2 A).

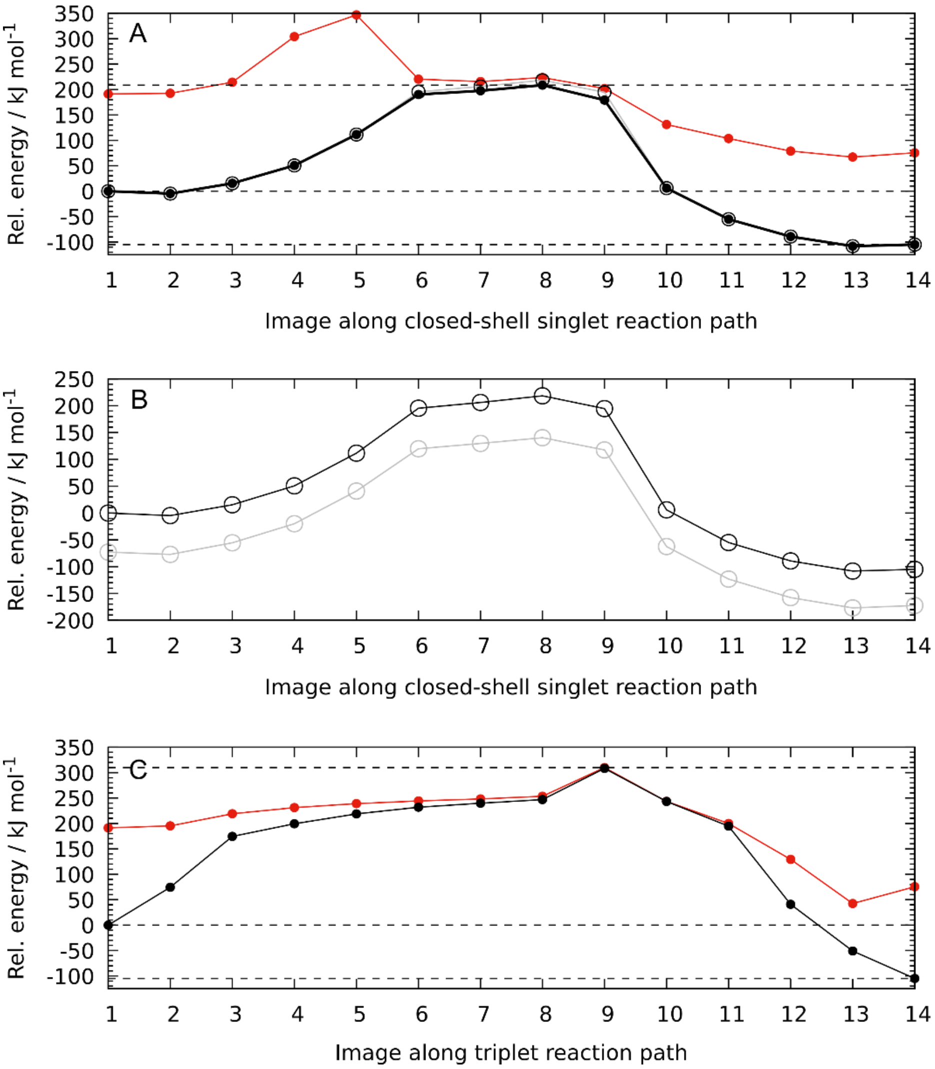

Figure S2: Reaction profiles, connecting singlet equilibria of **A(E$_A$E$_A$)** and **P(E$_A$E$_A$)**, **A**, along the closed-shell singlet minimum energy pathway, **B**, including solvent stabilization, and, **C**, along the triplet minimum energy pathway. Singlet energies are given in black (circles: closed-shell, dots: open-shell); solvent-stabilized closed-shell singlet energies are given in gray (**B**); triplet energies (open-shell: dots) are illustrated in red. All energies are calculated at the TPSSh/def2-SVP level of theory.

Furthermore, reaction profiles were simulated for the formation of all 13 possible products from the respective adducts at a reduced level of theory. Therefore, MEPs within the singlet multiplicity (closed-shell) were obtained by a locally developed program package[16] using the CI-NEB method and Grimme's semi-empirical GFN-xTB package.[17] The resulting PESs reveal that for the formation of **P(E$_A$E$_A$)** (Figure 3 A) the barrier is slightly lowered upon reduction of the level of theory (recall Figure 2 A). Both conceivable reaction pathways with correctly formed stereocenters – originating from **A(E$_A$E$_A$)** and **A(E$_A$Z$_B$)** – feature with approximately 170 kJ mol^{-1} almost identical activation energies, see Figure 3 A and Figure 3 D.

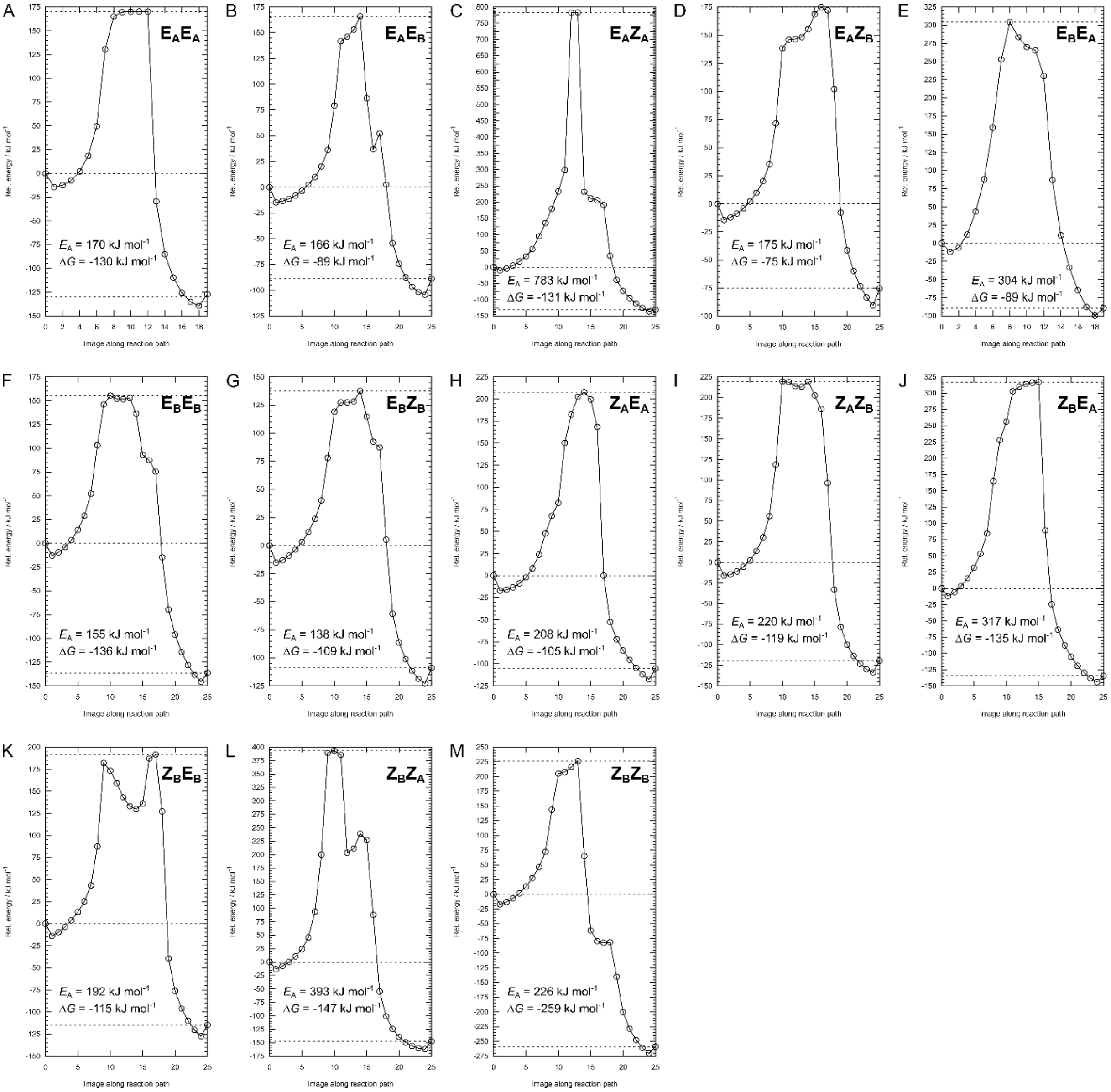

Figure S3. A – M Reaction profiles, connecting the respective adduct and product states (**3a**) originating from the two rotamers of **1a**, *E* (**E$_A$** and **E$_B$**) and *Z* (**Z$_A$** and **Z$_B$**). Closed-shell singlet ground state minimum energy pathways were obtained by CI-NEB and GFN-xTB. Relative Gibbs energies (ΔG in kJ mol^{-1}) and activation energies (E_A in kJ mol^{-1}) are indicated.

1.5 Structure Determinations

The intensity data for the compounds were collected on a Nonius KappaCCD diffractometer using graphite-monochromated Mo-K_α radiation. Data were corrected for Lorentz and polarization effects; absorption was taken into account on a semi-empirical basis using multiple-scans.[18]

The structures were solved by direct methods (SHELXS[19]) and refined by full-matrix least squares techniques against SHELXL-2018.[20] All hydrogen atoms of the compound **2d** and the hydrogen atoms bonded to the carbon atoms C1 to C4 of **1c**, **3a**, **4** and C3 of **2e** were located by difference Fourier synthesis and refined isotropically. All other hydrogen atoms were included at calculated positions with fixed thermal parameters.

The crystal of **3d**, and **3e** contains large voids, filled with disordered solvent molecules. The size of the voids are (475, and 292) Å^3/unit cell, respectively. Their contribution to the structure factors was secured by back-Fourier transformation using the SQUEEZE routine of the program PLATON[21] resulting in (215, and 80) electrons/unit cell, respectively. All non-hydrogen atoms were refined anisotropically.[21]

2 Molecular structures

Crystallographic data as well as structure solution and refinement details are summarized in Table S2. XP (SIEMENS Analytical X-ray Instruments, Inc.) was used for structure representations.

Crystallographic data (excluding structure factors) has been deposited with the Cambridge Crystallographic Data Centre as supplementary publication CCDC-1986392 for **1c**, CCDC-1986188 for **2c**, CCDC-1986189 for **2d**, CCDC-1986190 for **2e**, CCDC-1986191 for **3a**, CCDC-1986377 for **3d**, CCDC-1986192 for **3e**, and CCDC-1986193 for **4**. Copies of the data can be obtained free of charge on application to CCDC, 12 Union Road, Cambridge CB2 1EZ, UK [E- mail: deposit@ccdc.cam.ac.uk].

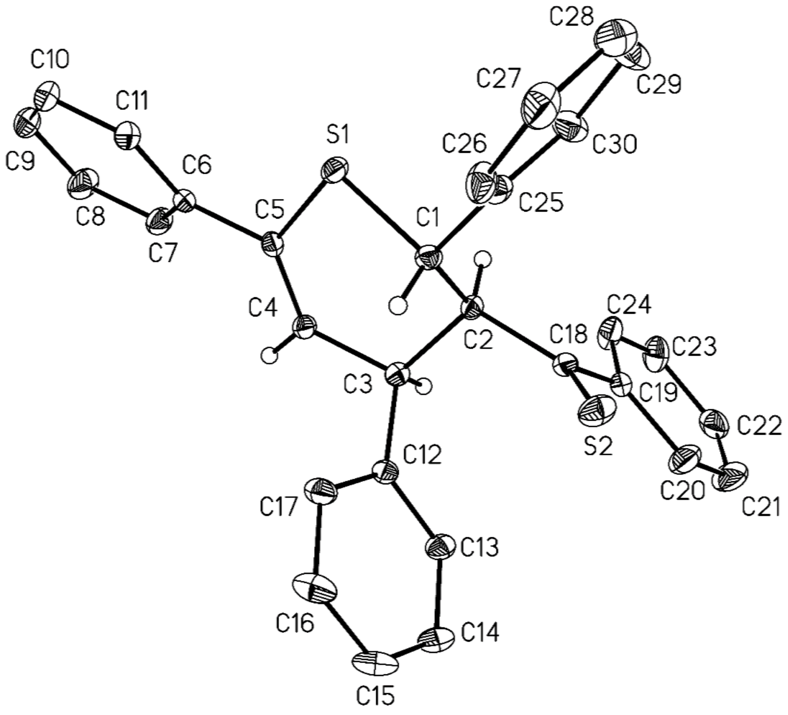

Figure S4. Molecular structure and atom labeling scheme of dimer B derived from **1c**. The ellipsoids represent a probability of 30 %, H atoms are shown with arbitrary radii. Selected bond lengths (Å): S(2)-C(18) 1.634(4).

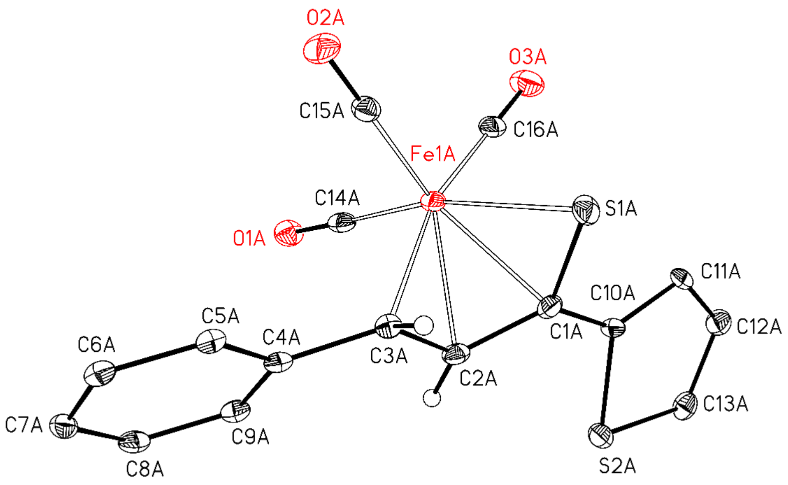

Figure S5. Molecular structure and atom labeling scheme of **2a**. The ellipsoids represent a probability of 30 %, H atoms are shown with arbitrary radii. Selected bond lengths (Å): S(1A)-C(1A) 1.741(3), C(1A)-C(2A) 1.421(4), C(2A)-C(3A) 1.420(4).

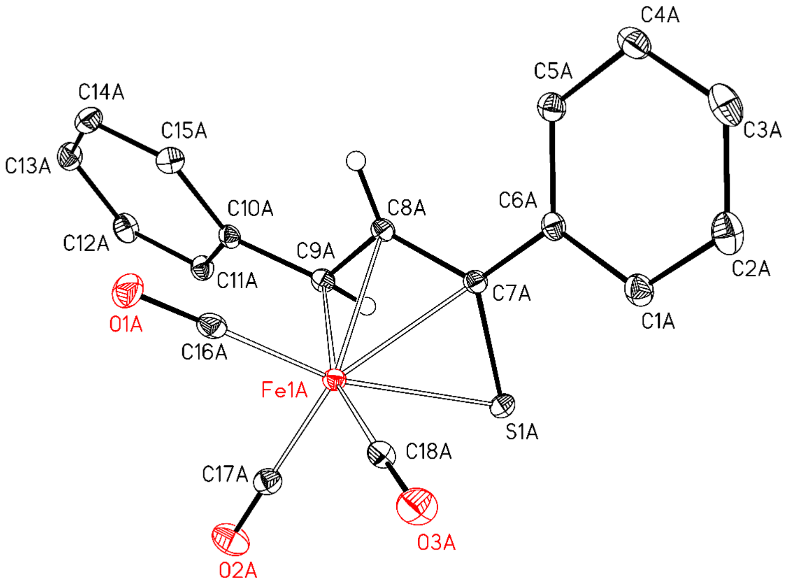

Figure S6. Molecular structure and atom labeling scheme of **2c**. The ellipsoids represent a probability of 30 %, H atoms are shown with arbitrary radii. Selected bond lengths (Å): S(1A)-C(7A) 1.745(2), C(7A)-C(8A) 1.418(3), C(8A)-C(9A) 1.427(3).

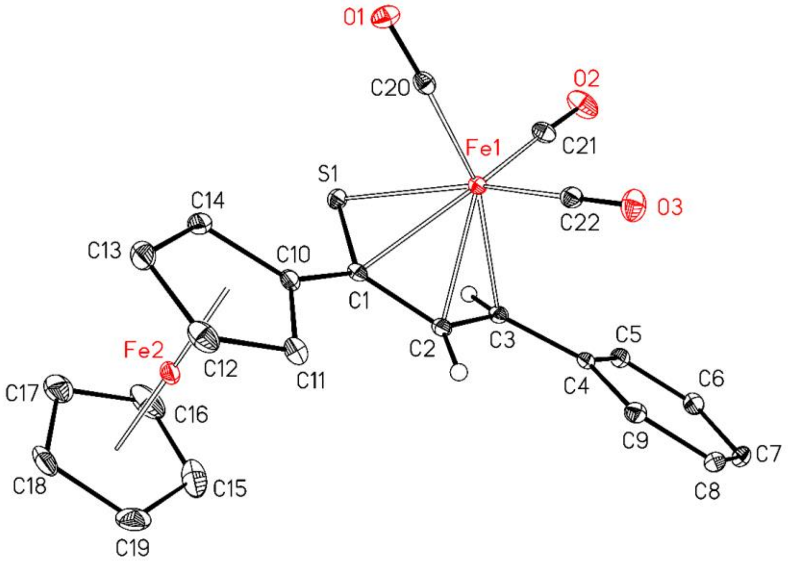

Figure S7. Molecular structure and atom labeling scheme of **2d**. The ellipsoids represent a probability of 30 %, H atoms are shown with arbitrary radii. Selected bond lengths (Å): S(1)-C(1) 1.7439(17), C(1)-C(2) 1.426(2), C(2)-C(3) 1.417(2).

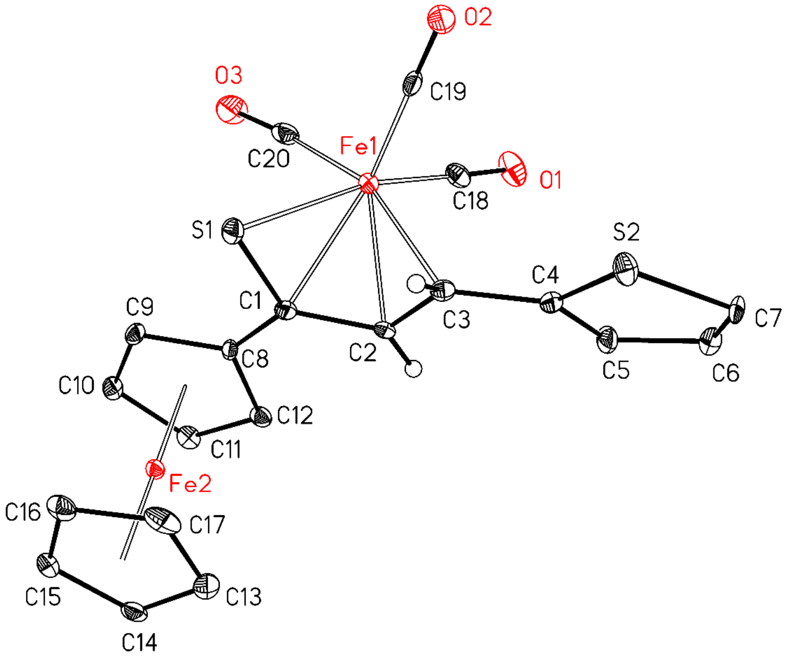

Figure S8. Molecular structure and atom labeling scheme of **2e**. The ellipsoids represent a probability of 30 %, H atoms are shown with arbitrary radii. Selected bond lengths (Å): S(1)-C(1) 1.739(4), C(1)-C(2) 1.416(5), C(2)-C(3) 1.412(5).

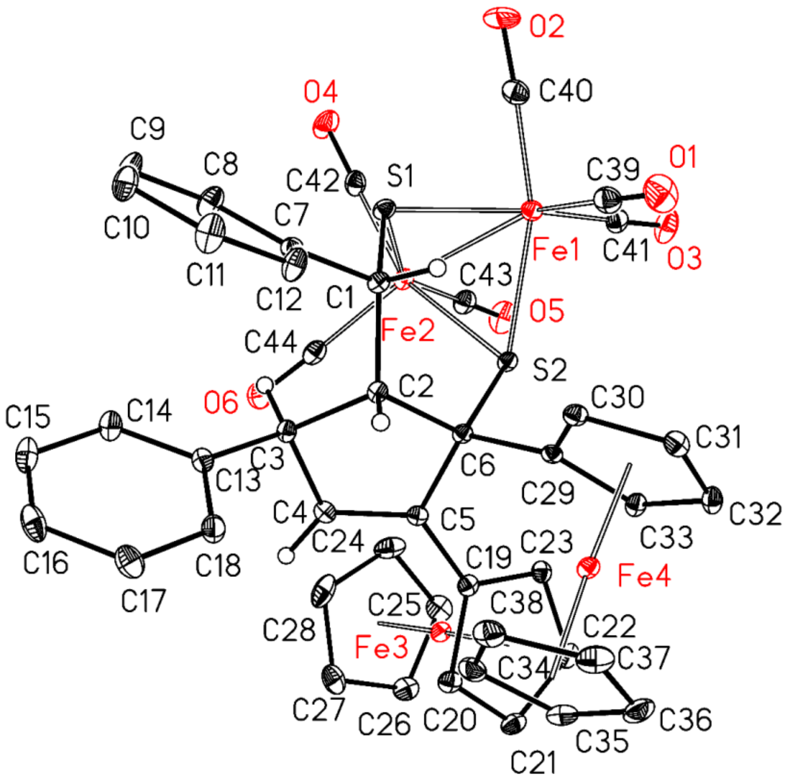

Figure S9. Molecular structure and atom labeling scheme of **3d**. The ellipsoids represent a probability of 30 %, H atoms are shown with arbitrary radii. Selected bond lengths (Å): Fe(1)-Fe(2) 2.5079(4), Fe(1)-S(1) 2.2495(6), Fe(1)-S(2) 2.2530(6), Fe(2)-S(1) 2.2727(6), Fe(2)-S(2) 2.2407(6), C(4)-C(5) 1.338(3); angles (°): S(1)-Fe(1)-S(2) 83.58(2), S(1)-Fe(1)-Fe(2) 56.761(17), S(2)-Fe(1)-Fe(2) 55.843(16), Fe(1)-S(1)-Fe(2) 67.439(15), Fe(2)-S(2)-Fe(1) 67.848(18).

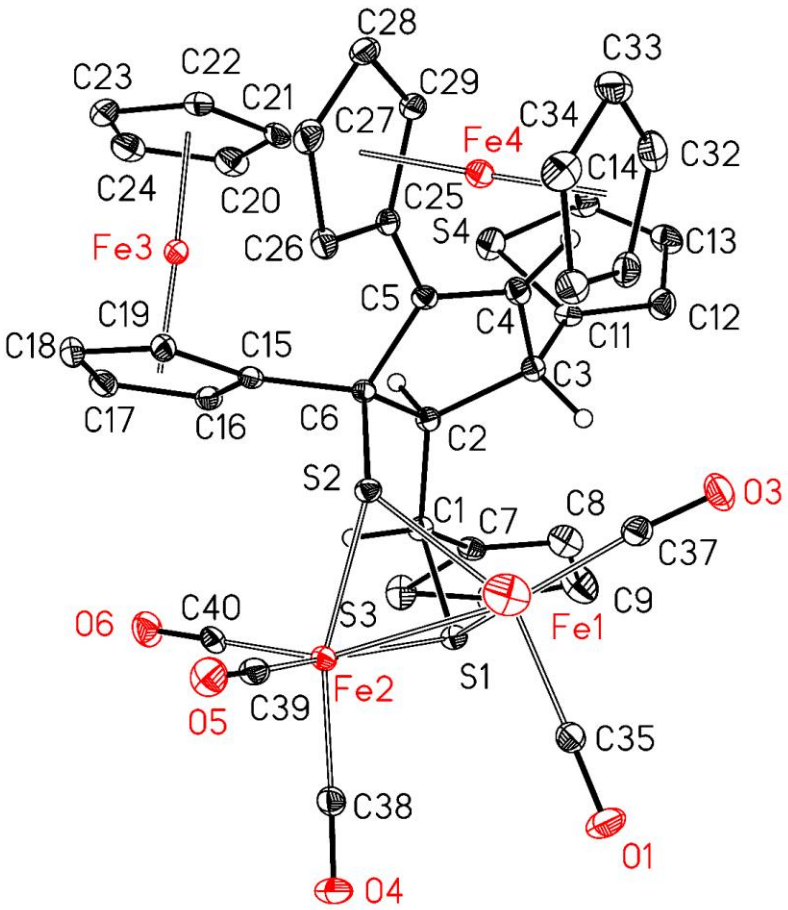

Figure S10. Molecular structure and atom labeling scheme of **3e**. The ellipsoids represent a probability of 30 %, H atoms are shown with arbitrary radii. Selected bond lengths (Å): Fe(1)-Fe(2) 2.5146(6), Fe(1)-S(1) 2.2495(9), Fe(1)-S(2) 2.2518(9), Fe(2)-S(1) 2.2485(9), Fe(2)-S(2) 2.2319(9), C(4)-C(5) 1.332(4); angles (°): S(1)-Fe(1)-S(2) 83.08(3), S(1)-Fe(1)-Fe(2) 55.99(2), S(2)-Fe(1)-Fe(2) 55.51(2), Fe(2)-S(1)-Fe(1) 67.98(3), Fe(2)-S(2)-Fe(1) 68.22(3).

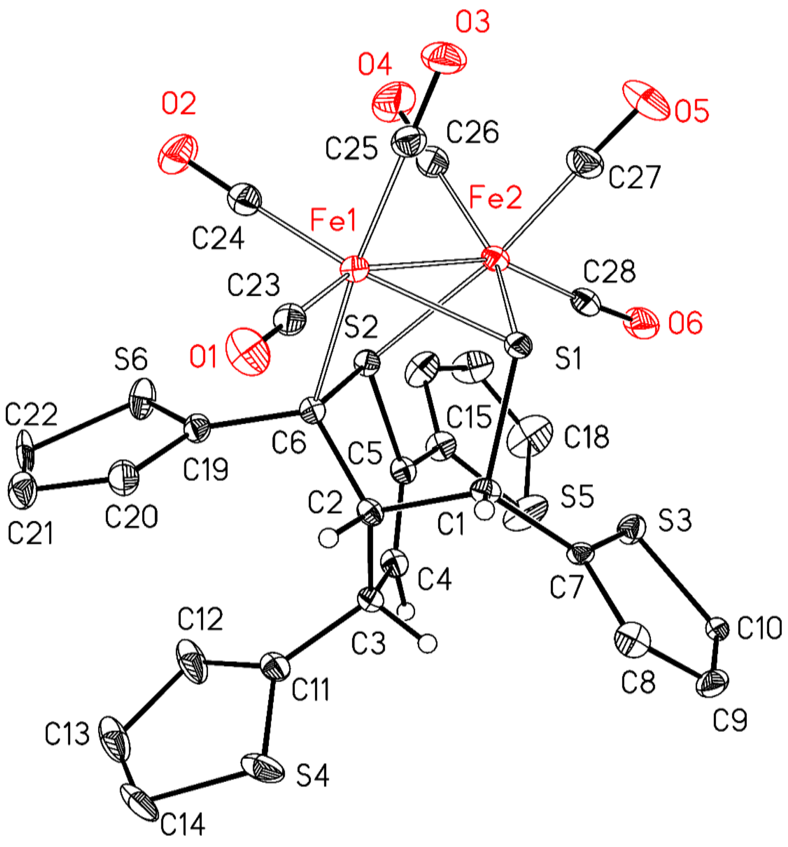

Figure S11. Molecular structure and atom labeling scheme of **4**. The ellipsoids represent a probability of 30 %, H atoms are shown with arbitrary radii. Selected bond lengths (Å): Fe(1)-Fe(2) 2.6365(6), Fe(1)-S(1) 2.2143(9), Fe(1)-C(6) 2.131(3), Fe(2)-S(1) 2.2430(9), Fe(2)-S(2) 2.2635(9), S(2)-C(5) 1.788(3); angles (°): C(6)-Fe(1)-S(1) 83.59(8), C(6)-Fe(1)-Fe(2) 82.67(8), S(1)-Fe(1)-Fe(2) 54.24(2), Fe(1)-S(1)-Fe(2) 72.52(3), S(1)-Fe(2)-S(2) 91.82(3), S(1)-Fe(2)-Fe(1) 53.23(2), S(2)-Fe(2)-Fe(1) 71.11(2).

Table S2: Crystal data and refinement details for the X-ray structure determinations.

Compound	**1c**	**2a**	**2c**	**2d**	**2e**	**3a**	**3d**	**3e**	**4**
formula	$C_{30}H_{24}S_2$	$C_{16}H_{10}FeO_3S_2$	$C_{18}H_{12}FeO_3S$	$C_{22}H_{16}Fe_2O_3S$	$C_{20}H_{14}Fe_2O_3S_2$	$C_{32}H_{20}Fe_2O_6S_4$	$C_{44}H_{32}Fe_4O_6S_2$ [*]	$C_{40}H_{28}Fe_4O6S_4$ [*]	$C_{28}H_{16}Fe_2O6S_6$
fw (g·mol^{-1})	448.61	370.21	364.19	472.11	478.13	740.42	944.21 [*]	956.26 [*]	752.47
T/°*C*	-140(2)	-140(2)	-140(2)	-140(2)	-140(2)	-140(2)	-140(2)	-140(2)	-140(2)
crystal system	monoclinic	orthorhombic	triclinic	monoclinic	monoclinic	monoclinic	triclinic	triclinic	orthorhombic
space group	P 2_1/c	P c a 2_1	P $\bar{1}$	P 2_1	P 2_1	P 2_1/c	P $\bar{1}$	P $\bar{1}$	P n n a
a/ Å	17.4175(8)	7.36270(10)	9.2326(2)	7.5337(1)	7.2300(2)	12.4609(2)	11.7317(3)	9.2923(3)	21.4814(4)
b/ Å	13.2987(6)	10.5736(2)	11.1911(2)	11.2202(3)	19.2587(5)	17.2330(2)	12.0885(3)	11.6337(3)	17.7378(3)
c/ Å	10.2512(3)	38.7371(8)	16.5850(4)	11.2731(3)	7.4499(2)	14.8589(2)	18.1949(4)	19.5942(6)	16.0989(3)
α/°	90	90	70.469(1)	90	90	90	105.634(1)	102.709(1)	90
β/°	99.875(2)	90	89.609(1)	97.348(1)	117.110(1)	105.669(1)	95.573(2)	96.186(2)	90
γ/°	90	90	78.848(1)	90	90	90	111.121(1)	94.932(2)	90
V/Å^3	2339.31(17)	3015.69(9)	1581.41(6)	945.09(4)	923.36(4)	3072.21(7)	2263.15(10)	2041.10(11)	6134.21(19)
Z	4	8	4	2	2	4	2	2	8
ρ (g·cm^{-3})	1.274	1.631	1.530	1.659	1.720	1.601	1.386 [*]	1.556 [*]	1.630
μ (cm^{-1})	2.43	12.84	10.96	16.66	18.15	12.61	13.91 [*]	16.42 [*]	13.95
measured data	25813	37045	12153	13468	11466	24271	30753	25005	43926
data with I > 2σ(I)	4415	6607	6457	4267	3905	6286	9156	7335	5575
unique data (R_{int})	5348/0.0825	6761/0.0377	7129/0.0259	4305/0.0207	4170/0.0340	7045/0.0325	10314/0.0261	9235/0.0343	7027/0.0973
w*R*$_2$ (all data, on F^2)$^{a)}$	0.1689	0.1058	0.0773	0.0422	0.0755	0.0639	0.0876	0.0928	0.1000
R$_1$ (*I* > 2σ(*I*)) $^{a)}$	0.0843	0.0377	0.0357	0.0181	0.0352	0.0294	0.0333	0.0450	0.0498
S $^{b)}$	1.238	1.163	1.043	1.058	1.136	1.062	1.058	1.071	1.106
Res. dens./e·Å^{-3}	0.894/-0.323	0.808/-0.684	0.498/-0.528	0.285/-0.230	0.316/-0.326	0.351/-0.225	0.423/-0.347	0.592/-0.473	0.730/-0.373
Flack-parameter	-	0.399(18)	-	0.001(5)	-0.021(13)	multi-scan	multi-scan	multi-scan	multi-scan
absorpt method	multi-scan	multi-scan	multi-scan	multi-scan	multi-scan	0.7159/0.7456	0.7015/0.7456	0.6980/0.7456	0.6758/0.7456
absorpt corr $T_{min/max}$	0.6671/0.7456	0.6600/0.7457	0.7027/0.7456	0.6983/0.7456	0.6908/0.7456	1986191	-1986377	1986192	1986193
CCDC No.	1986392	1986187	1986188	1986189	1986190	**3a**	**3d**	**3e**	**4**

[*] derived parameters do not contain the contribution of the disordered solvent.

a) Definition of the *R* indices: $R_1 = (\Sigma||F_o|-|F_c||)/\Sigma|F_o|$;

$wR_2 = \{\Sigma[w(F_o^2-F_c^2)^2]/\Sigma[w(F_o^2)^2]\}^{1/2}$ with $w^{-1} = \sigma^2(F_o^2) + (aP)^2+bP$; $P = [2F_c^2 + Max(F_O^2]/3$;

b) $s = \{\Sigma[w(F_o^2-F_c^2)^2]/(N_o-N_p)\}^{1/2}$.

3 Cyclic voltammetry

Figure S12 shows the cyclic voltammetry of complexes **3a-e** in CH_2Cl_2-[*n*-Bu_4N][BF_4] solutions at a scan rate of 0.2 V s^{-1}.

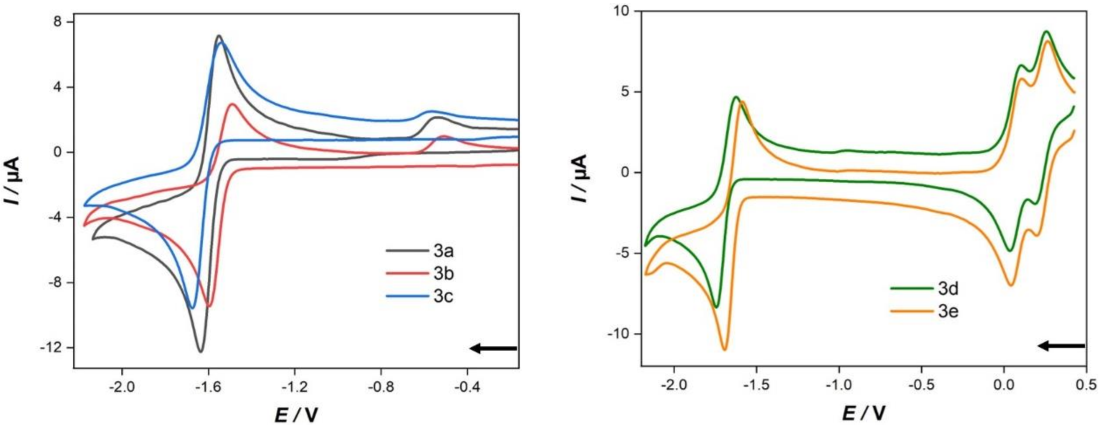

Figure S12. Cyclic voltammetry of 1.0 mM complexes **3a-c** (left) and complexes **3d-e** (right) in CH_2Cl_2-[*n*-Bu_4N][BF_4] (0.1 M) solutions at 0.2 V s^{-1} scan rate. The arrows indicate the scan direction. The potentials *E* are given in V and referenced to the Fc^+/Fc couple.

The cyclic voltammetry of complexes **3a-e** at a scan rate of 0.2 V s^{-1} exhibit quasi-reversible peaks at E_{pc} = (-1.64, -1.60, -1.67, -1.74 and -1.69) V, respectively, in their cathodic region, which are all ascribed to the reduction process of Fe^IFe^I to Fe^IFe^0 similar to their reported analogue complexes.[22] It can be noticed from Figure S12 that complex **3d** shows a more negative reduction potential in comparison to complexes **3a-c** and **3e**. This indicates that the electron density of the iron core of **3d** is higher than those of **3a-c** and **3e**, as consistent with the IR data. On initiating the electrochemical scan in the anodic direction of complexes **3d** and **3e**, two quasi-reversible anodic events at $E_{1/2}$ = 0.07 V and 0.22 V for complex **3d** and at $E_{1/2}$ = 0.08 V and 0.23 V for complex **3e** were observed. These oxidation peaks are corresponding to one-electron transfer of the ferrocene ligands.

The electrocatalytic ability of complexes **3a-e** toward hydrogen production were investigated in the presence of various concentrations of acetic acid (AcOH). However, their electrocatalytic behaviors are almost identical. Therefore, complex **3d** is illustrated in Figure S13 exemplarily. The addition of different equivalents of AcOH to a solution of complex **3d** causes a small shift of its primary reduction peak to less negative potential together with the disappearance of its anodic peak in the reverse scan (Figure 13), which is a typical observation when protonation of a reduced species takes place.[23] At more reducing potentials, in the vicinity of -2.00 V to -2.30 V, the voltammetric profile of complex **3d** shows a reduction peak with a current that increases in response to the systematic increase in the acid concentration, indicating a catalytic proton reduction.

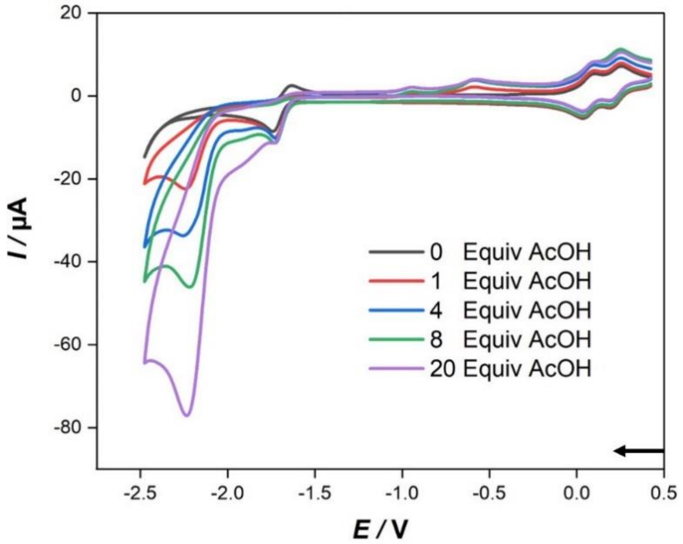

Figure S13. Cyclic voltammetry of 1.0 mM of complex **3d** in CH_2Cl_2-[*n*-Bu_4N][BF_4] (0.1 M) solutions at 0.2 V s^{-1} in the presence of various concentrations of AcOH. The arrows indicate the scan directions. The potentials *E* are given in V and referenced to the Fc^+/Fc couple.

4 NMR spectra

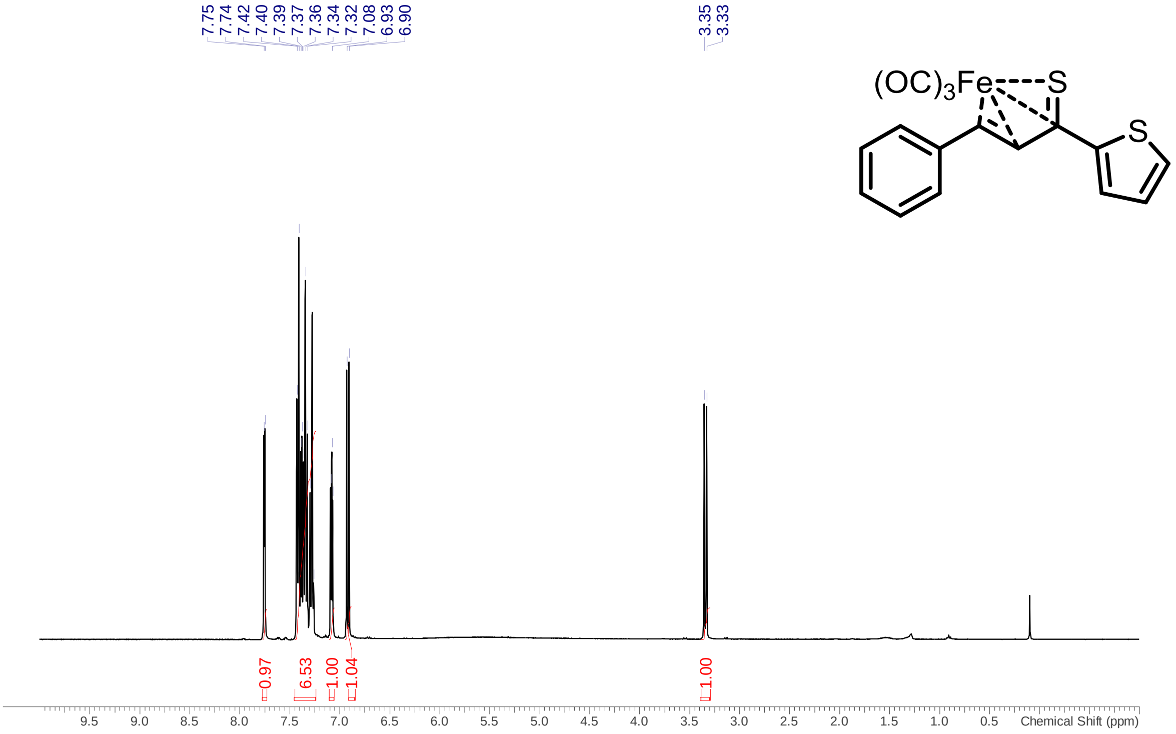

^{1}H NMR spectrum ($CDCl_3$, 400 MHz) of **2a**.

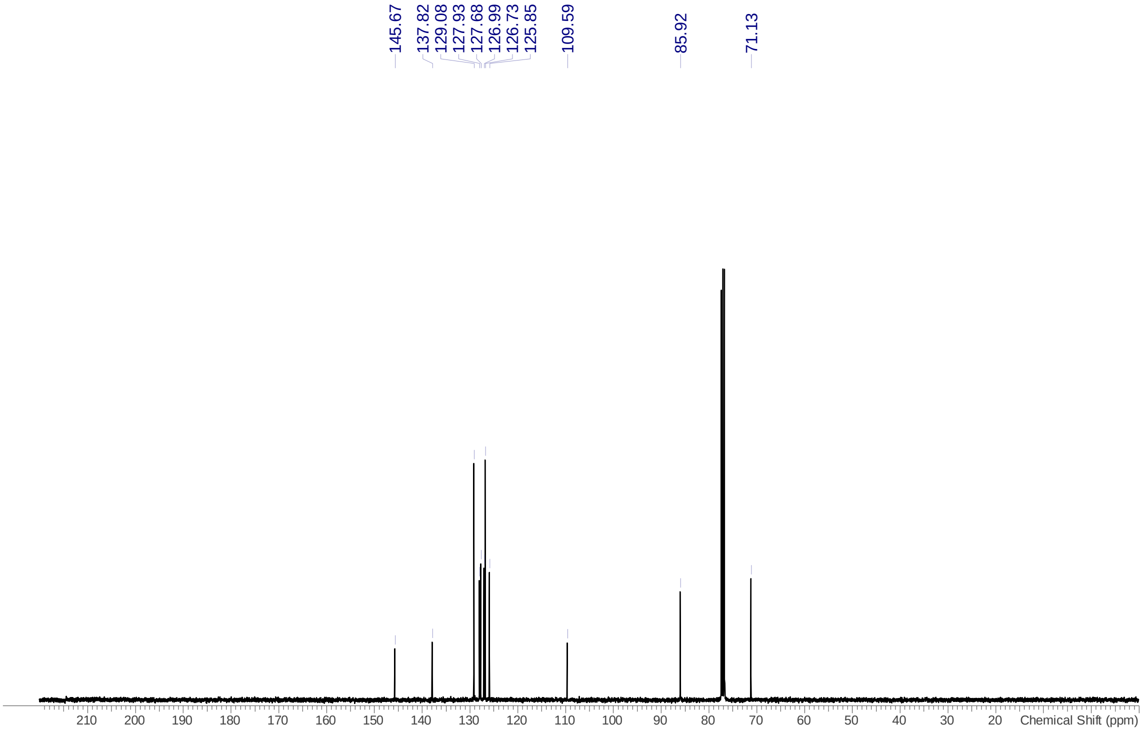

^{13}C NMR spectrum ($CDCl_3$, 101 MHz) of **2a**.

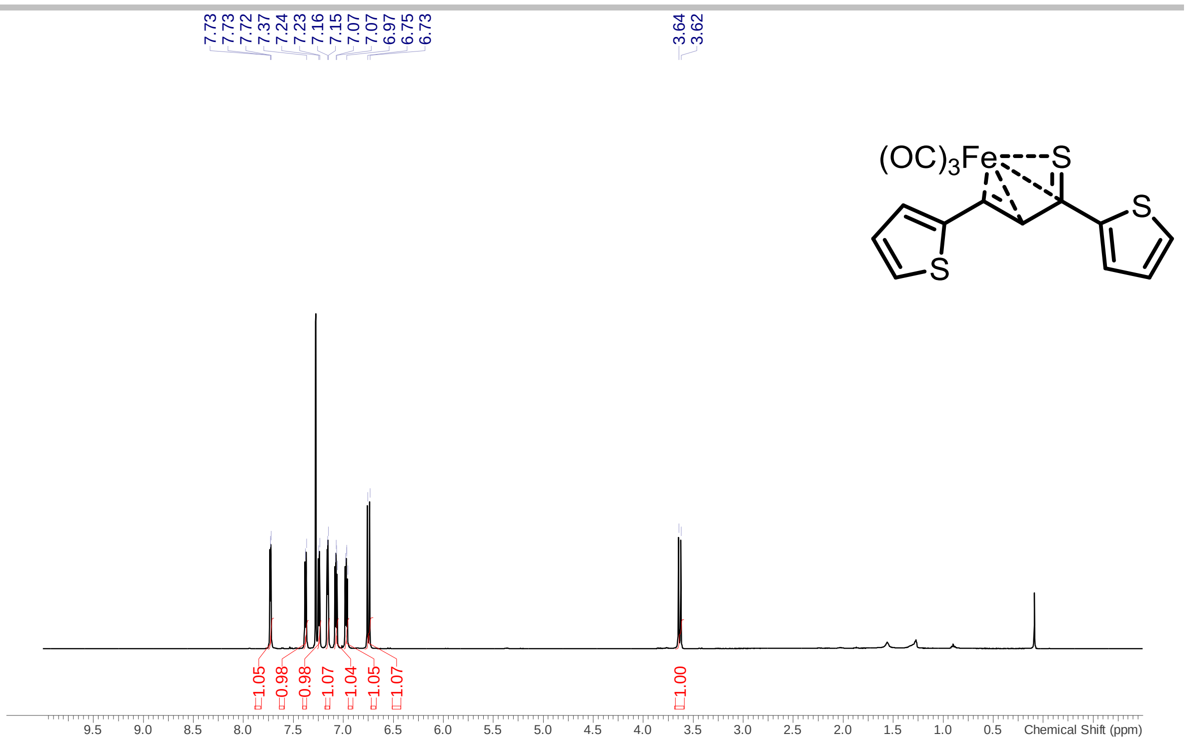

^{1}H NMR spectra ($CDCl_3$, 400 MHz) of **2b**.

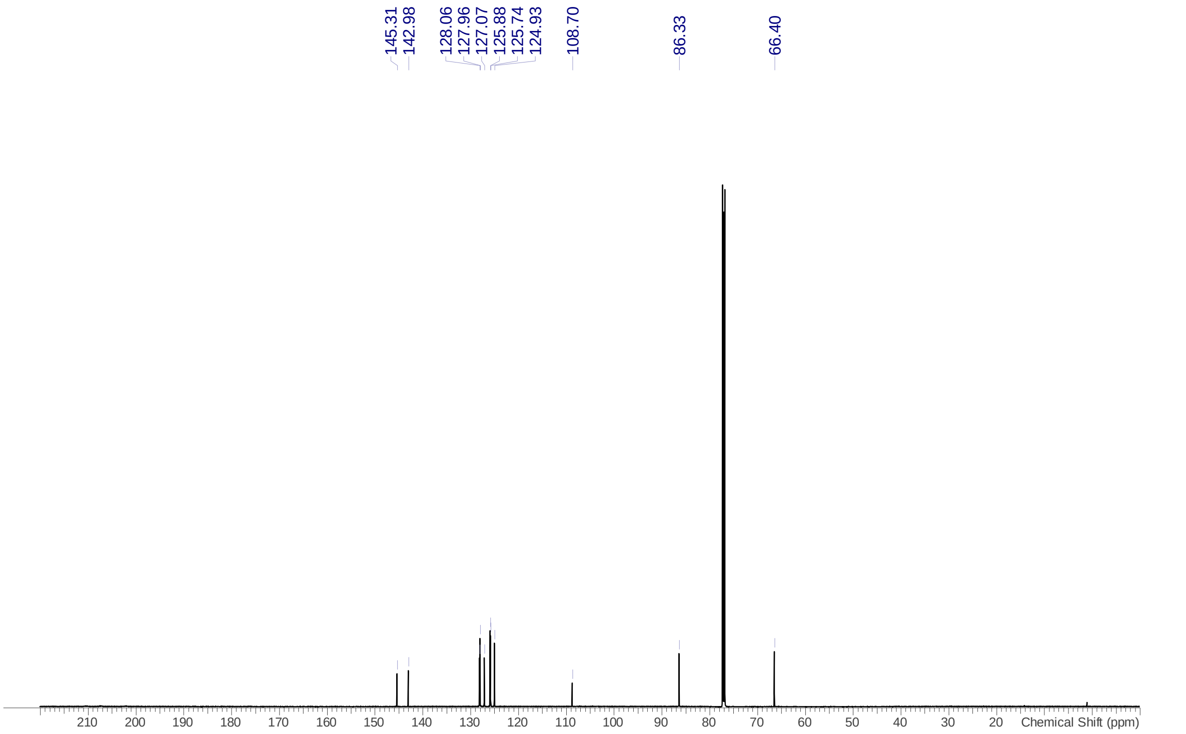

^{13}C NMR spectra ($CDCl_3$, 151 MHz) of **2b**.

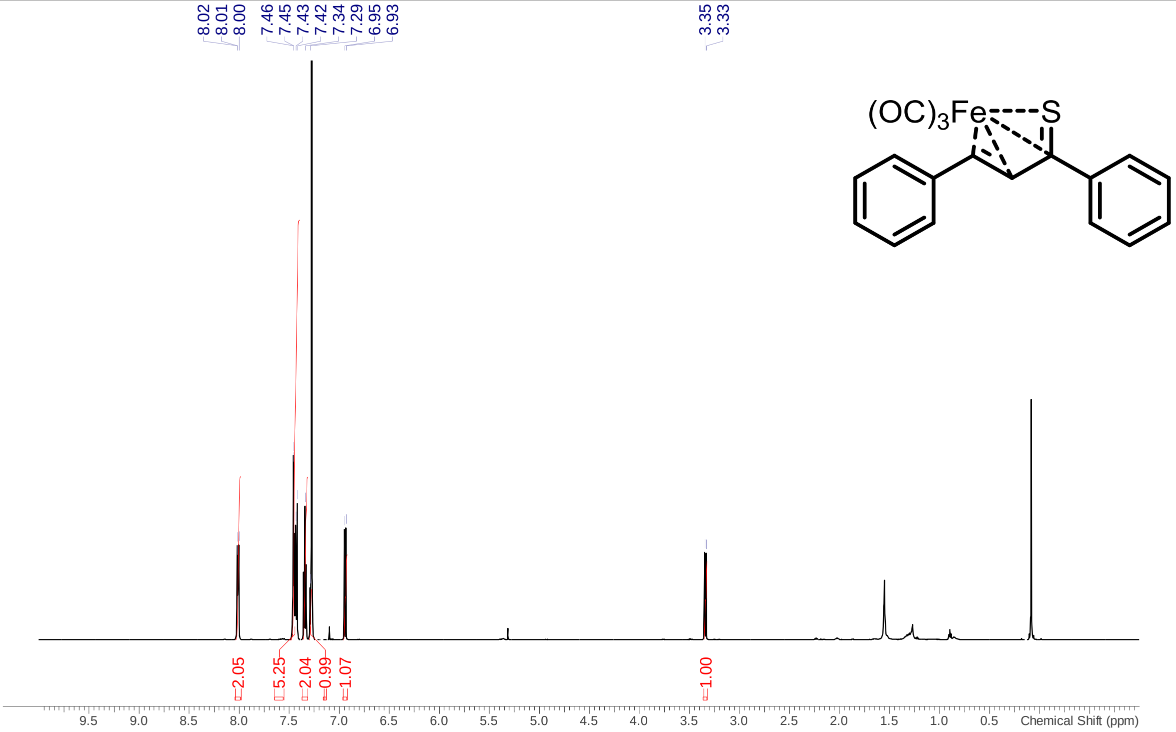

^{1}H NMR spectra ($CDCl_3$, 600 MHz) of **2c**.

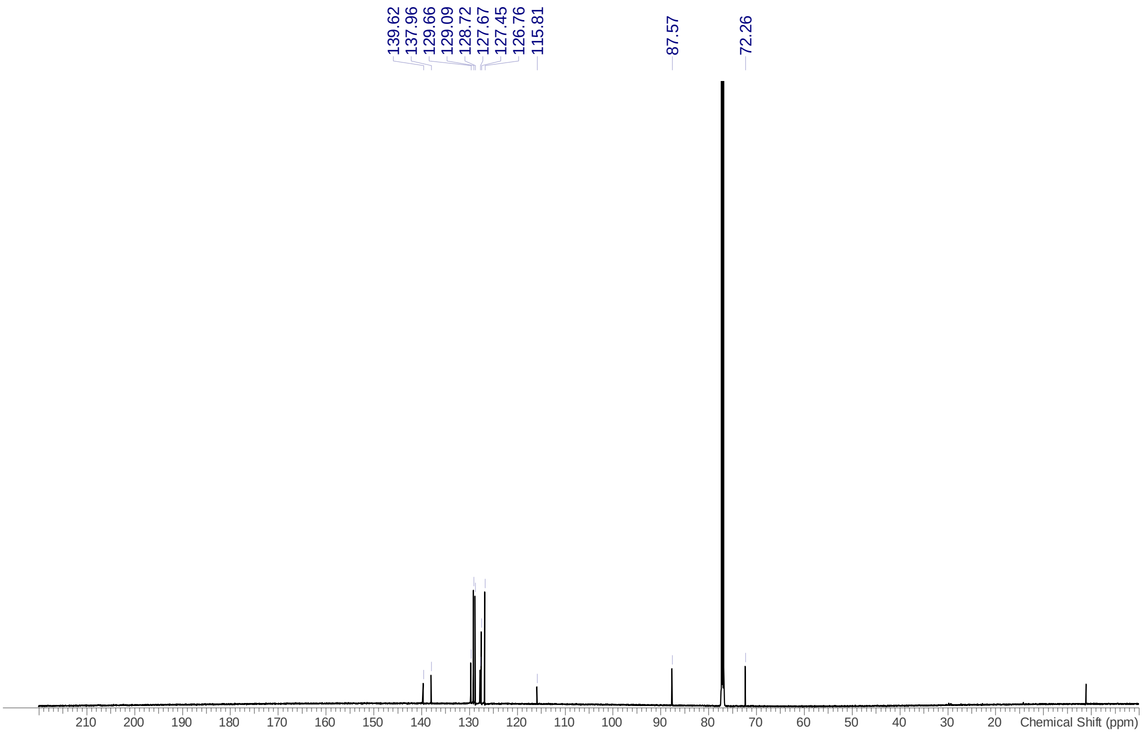

^{13}C NMR spectra ($CDCl_3$, 151 MHz) of **2c**.

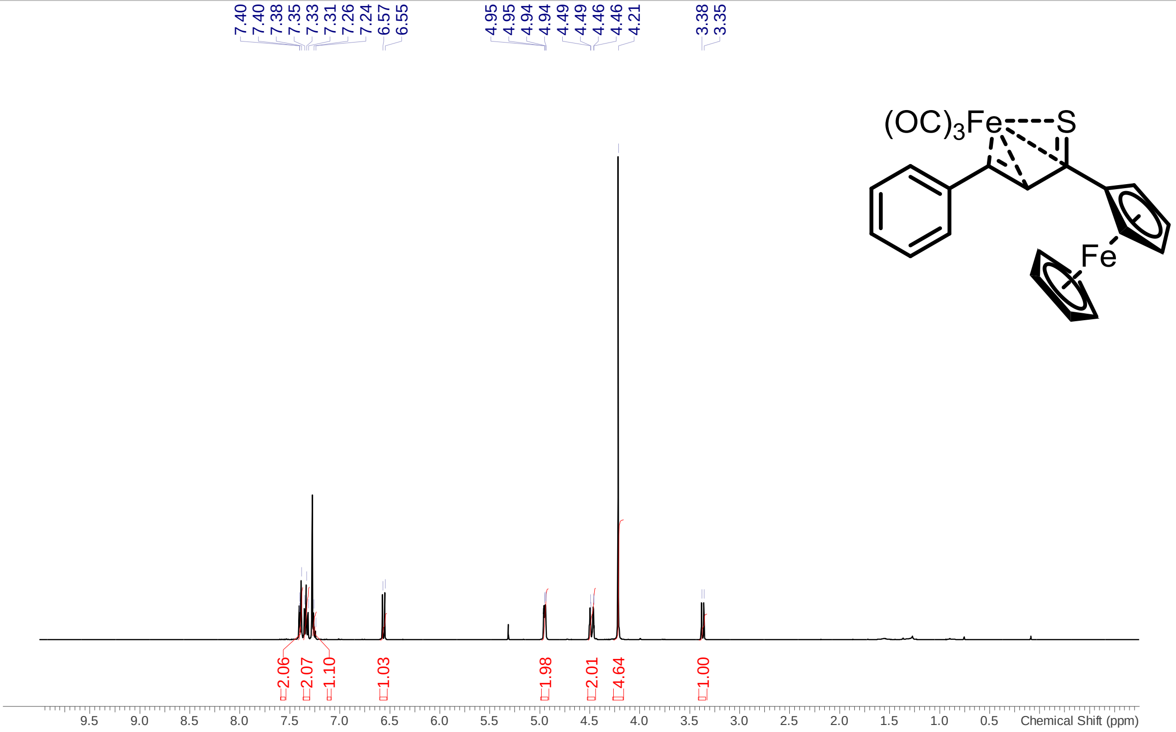

^{1}H NMR spectra ($CDCl_3$, 400 MHz) of **2d**.

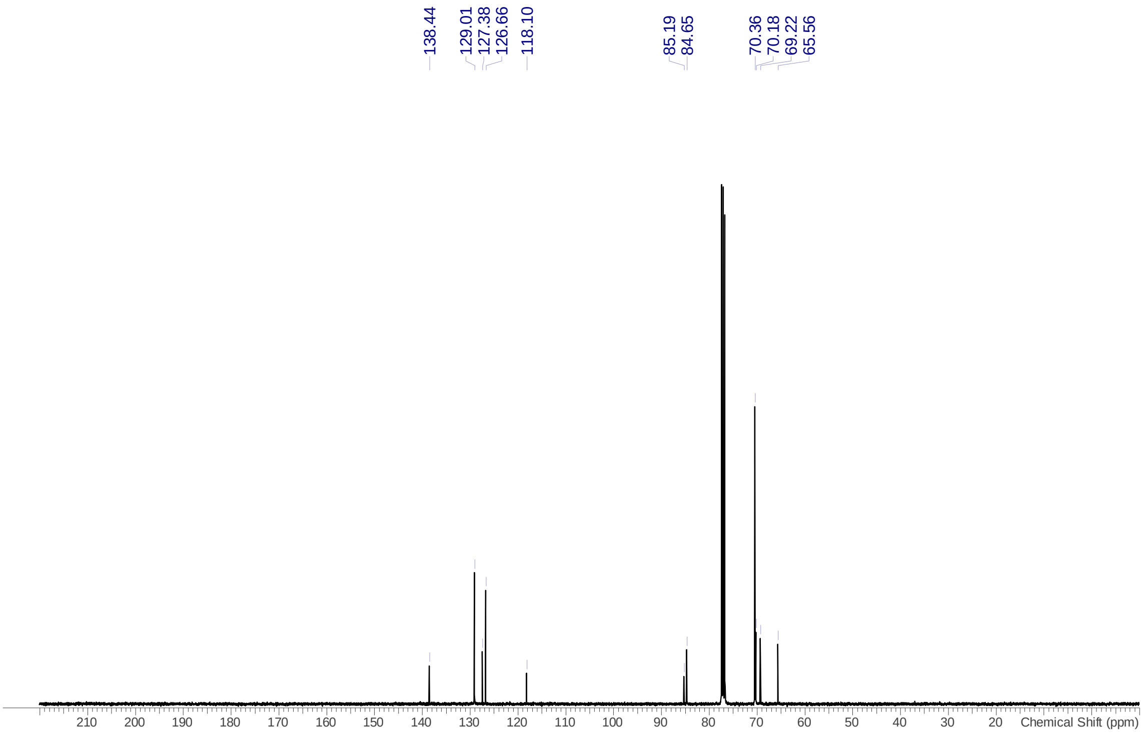

^{13}C NMR spectra ($CDCl_3$, 101 MHz) of **2d**.

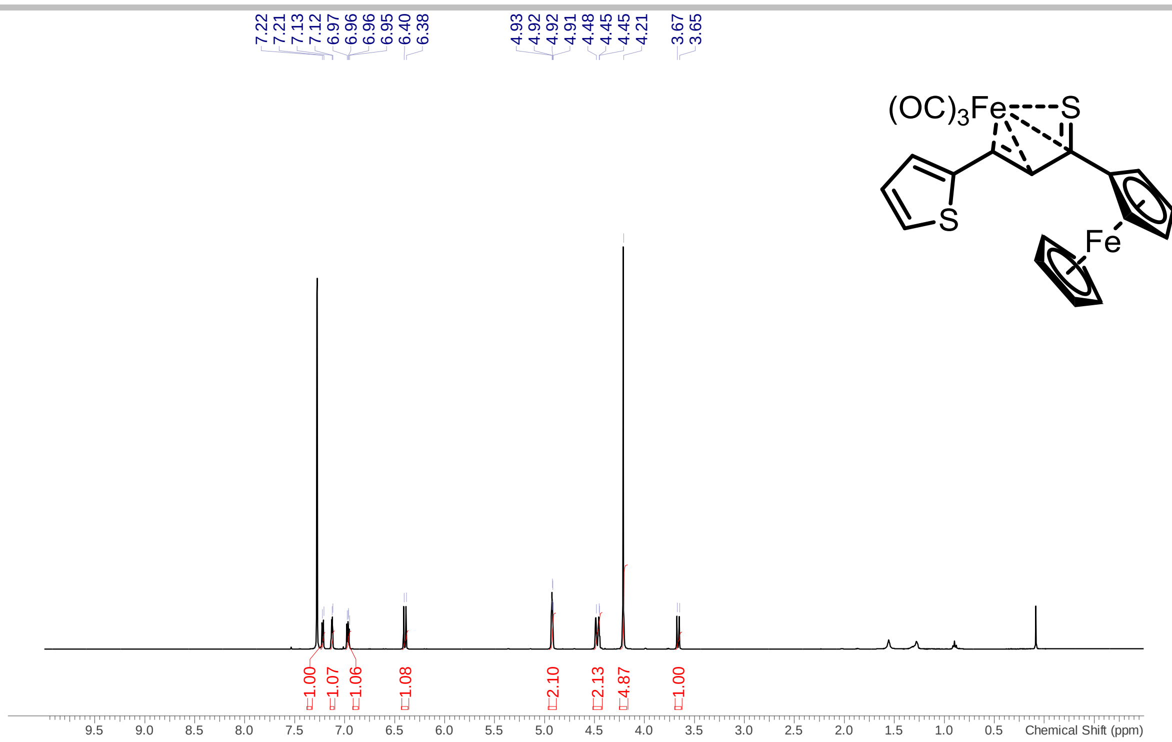

^{1}H NMR spectra ($CDCl_3$, 600 MHz) of **2e**.

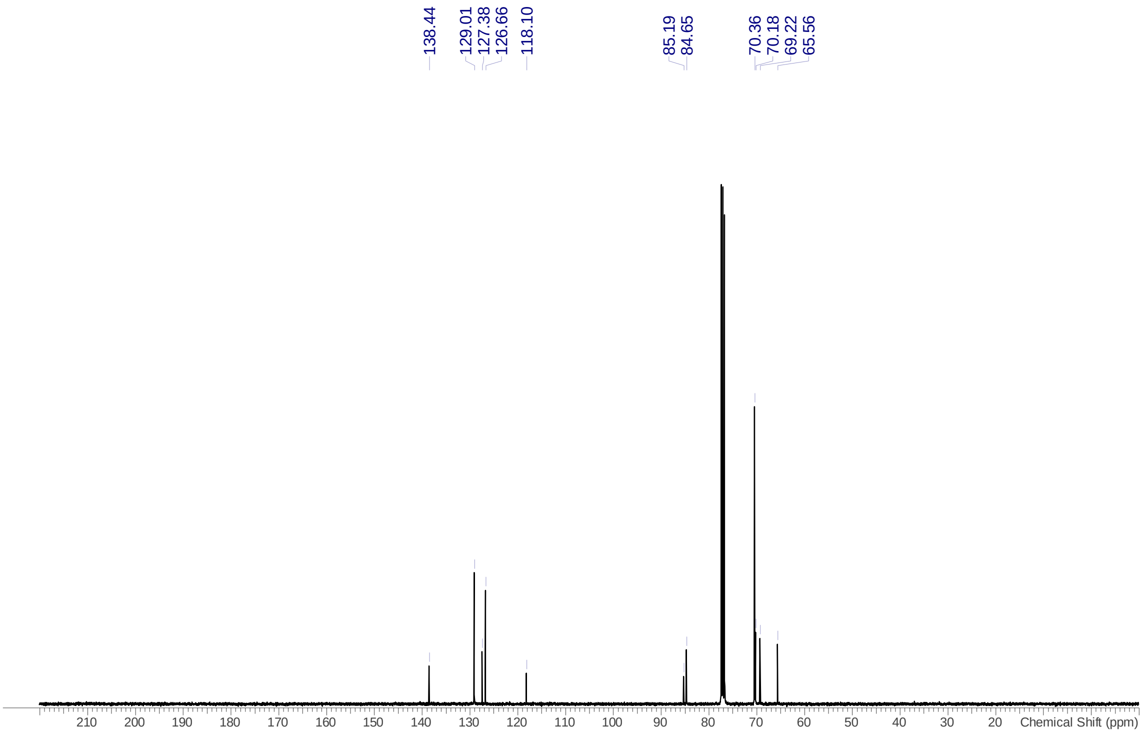

^{13}C NMR spectra ($CDCl_3$, 151 MHz) of **2e**.

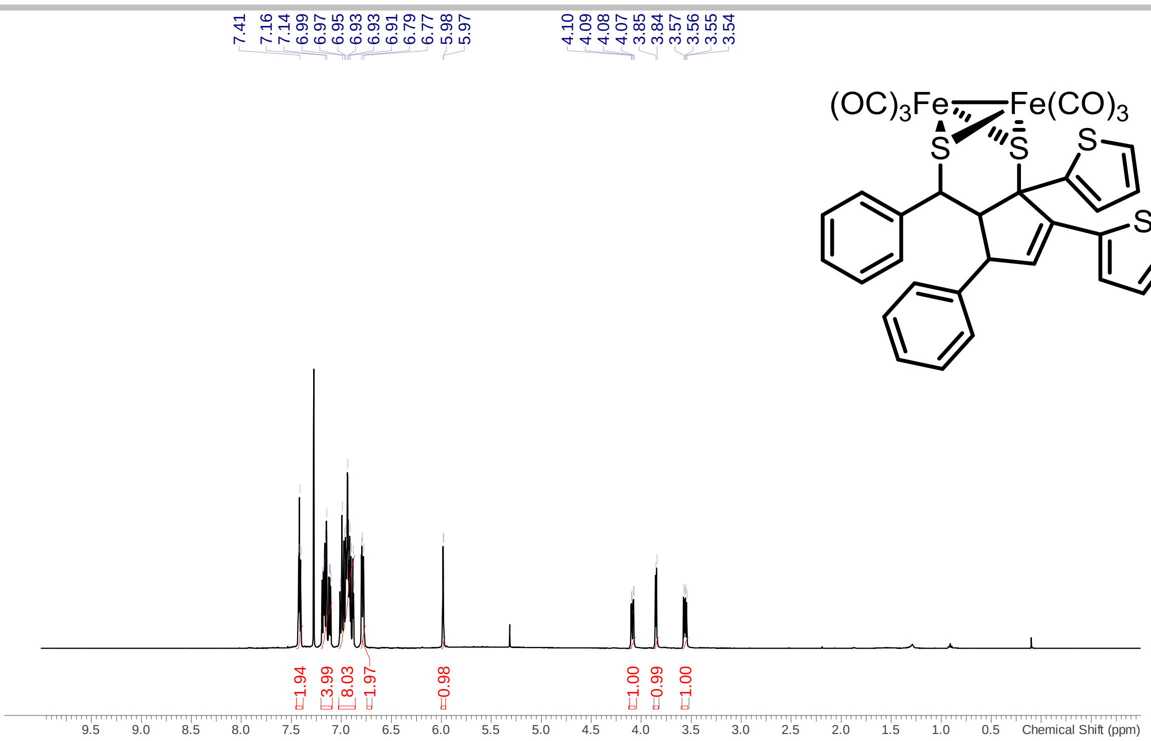

^{1}H NMR spectra ($CDCl_3$, 400 MHz) of **3a**.

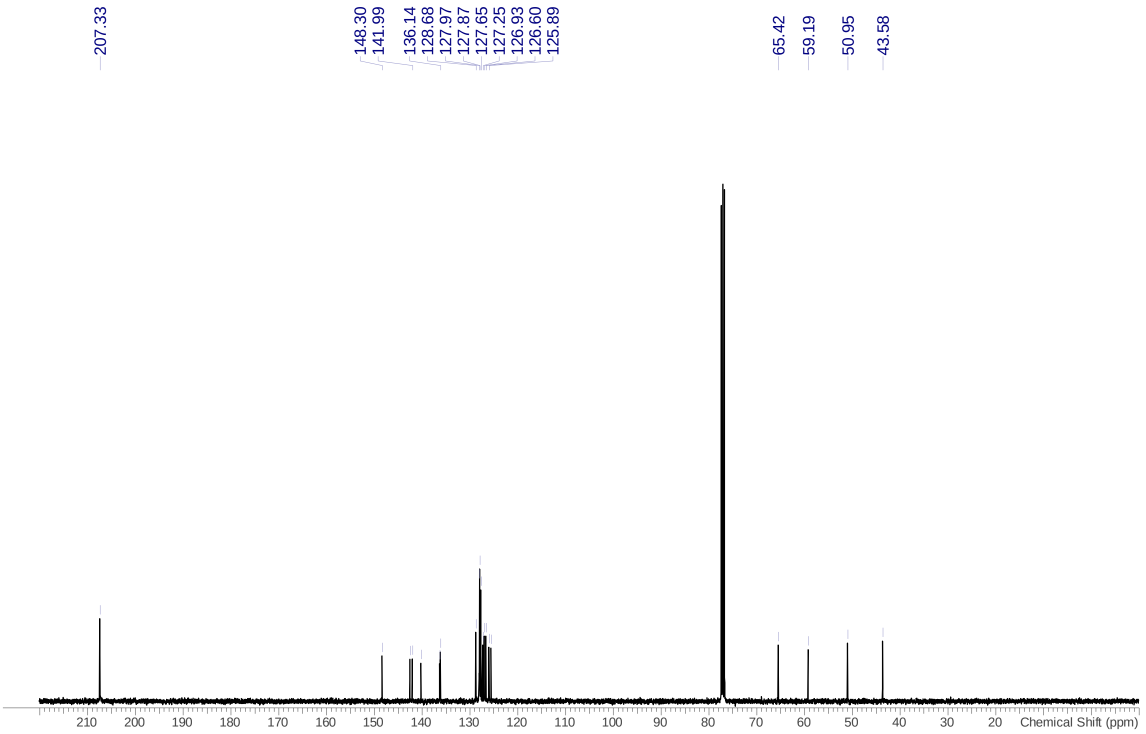

^{13}C NMR spectra ($CDCl_3$, 101 MHz) of **3a**.

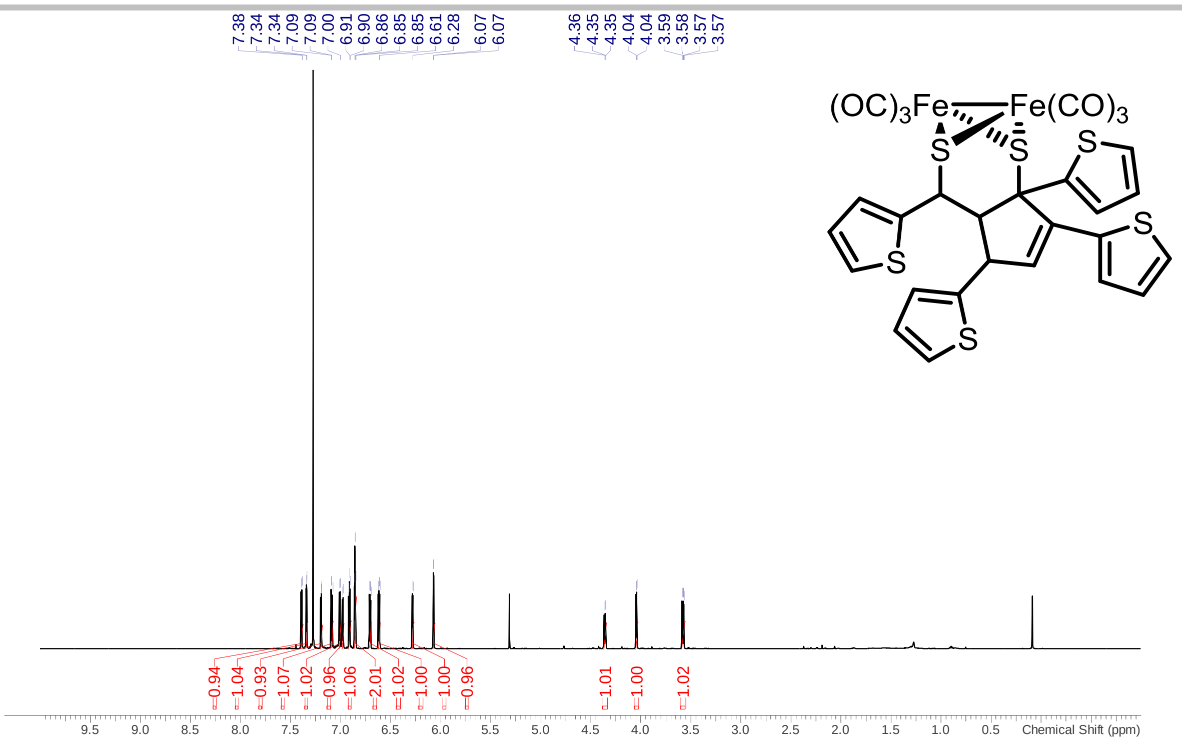

^{1}H NMR spectra ($CDCl_3$, 600 MHz) of **3b**.

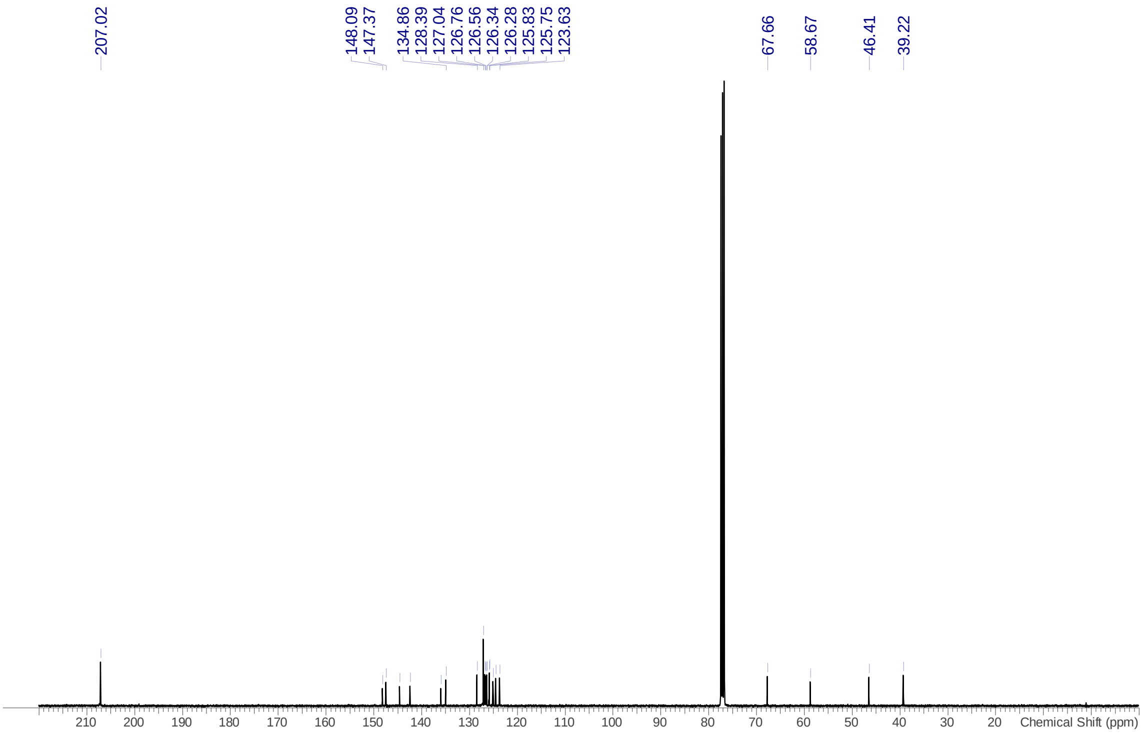

^{13}C NMR spectra ($CDCl_3$, 101 MHz) of **3b**.

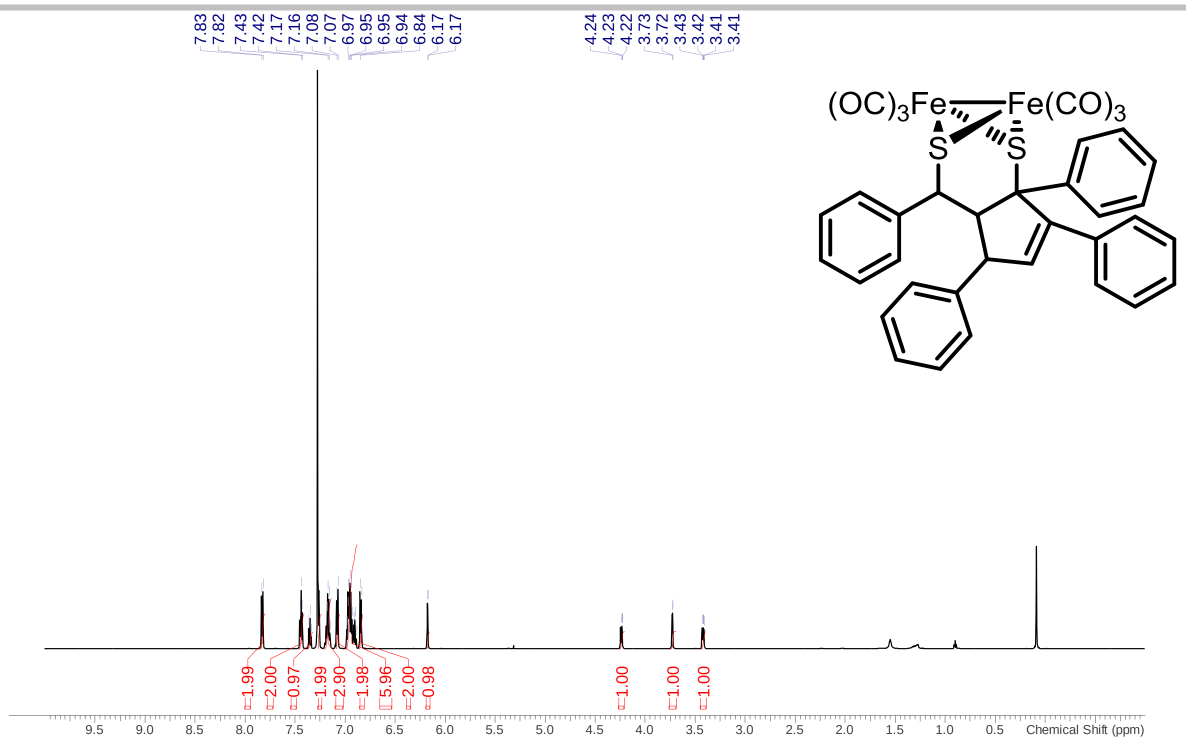

^{1}H NMR spectra ($CDCl_3$, 600 MHz) of **3c**.

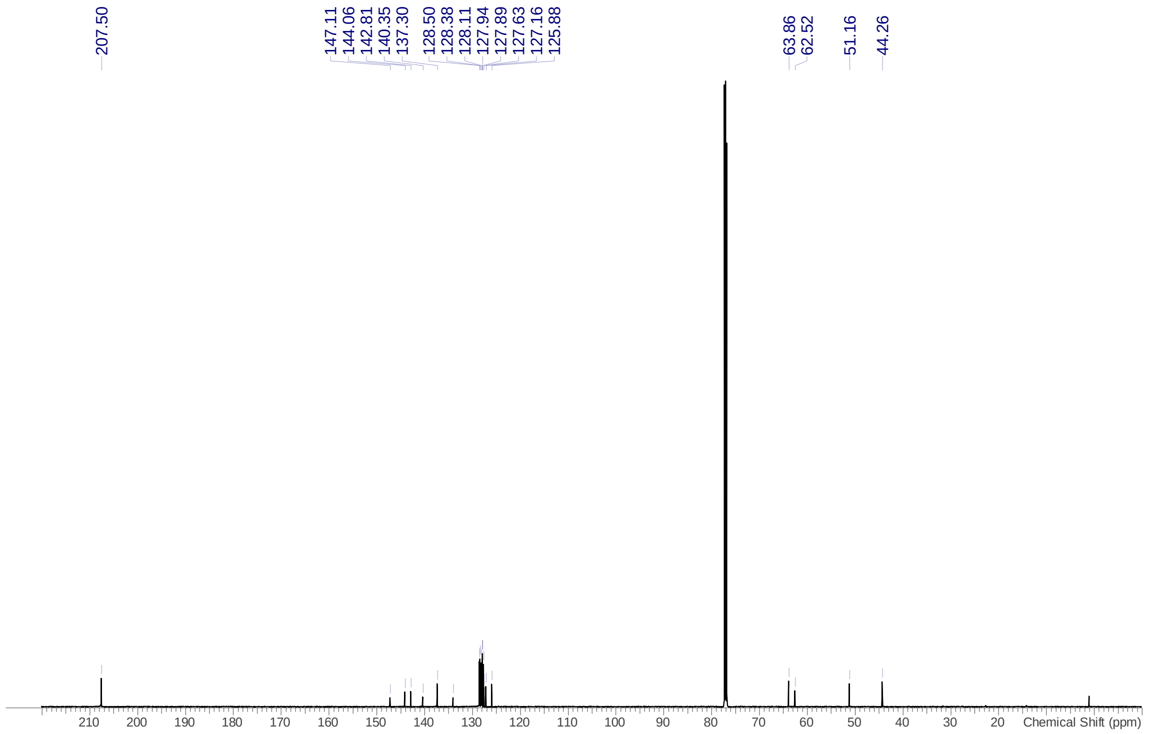

^{13}C NMR spectra ($CDCl_3$, 151 MHz) of **3c**.

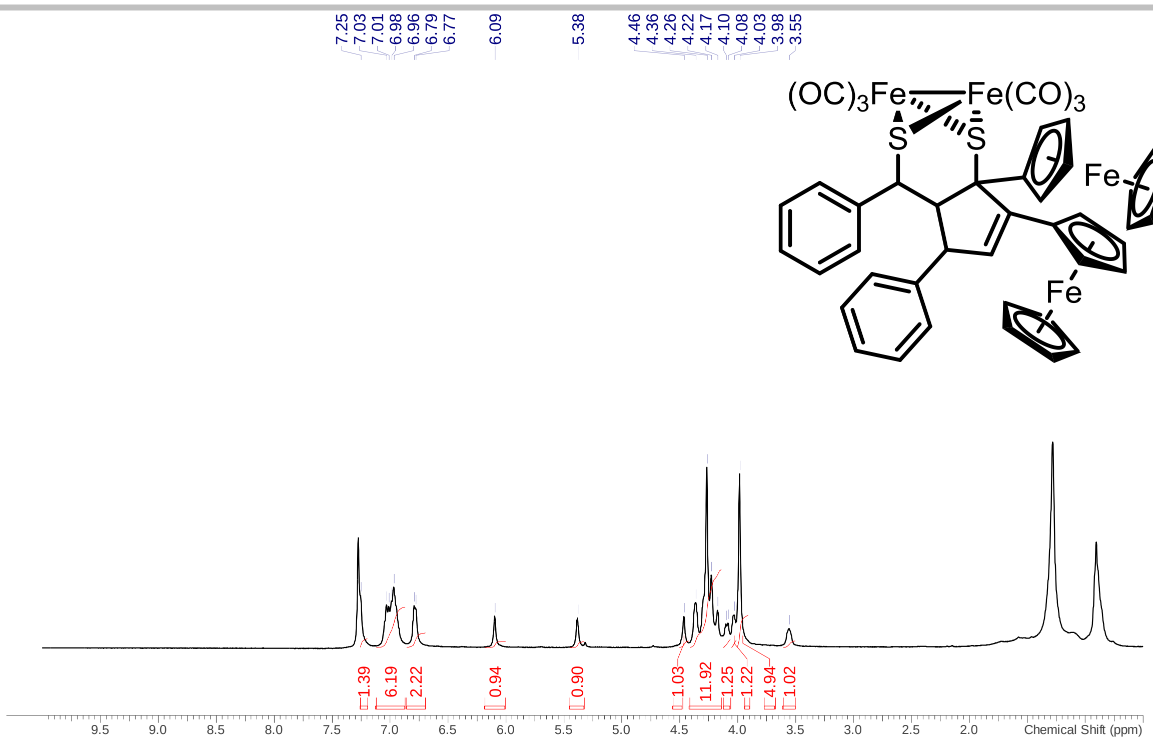

^{1}H NMR spectra ($CDCl_3$, 400 MHz) of **3d** (*n*-hexane in crystals, see elemental analysis).

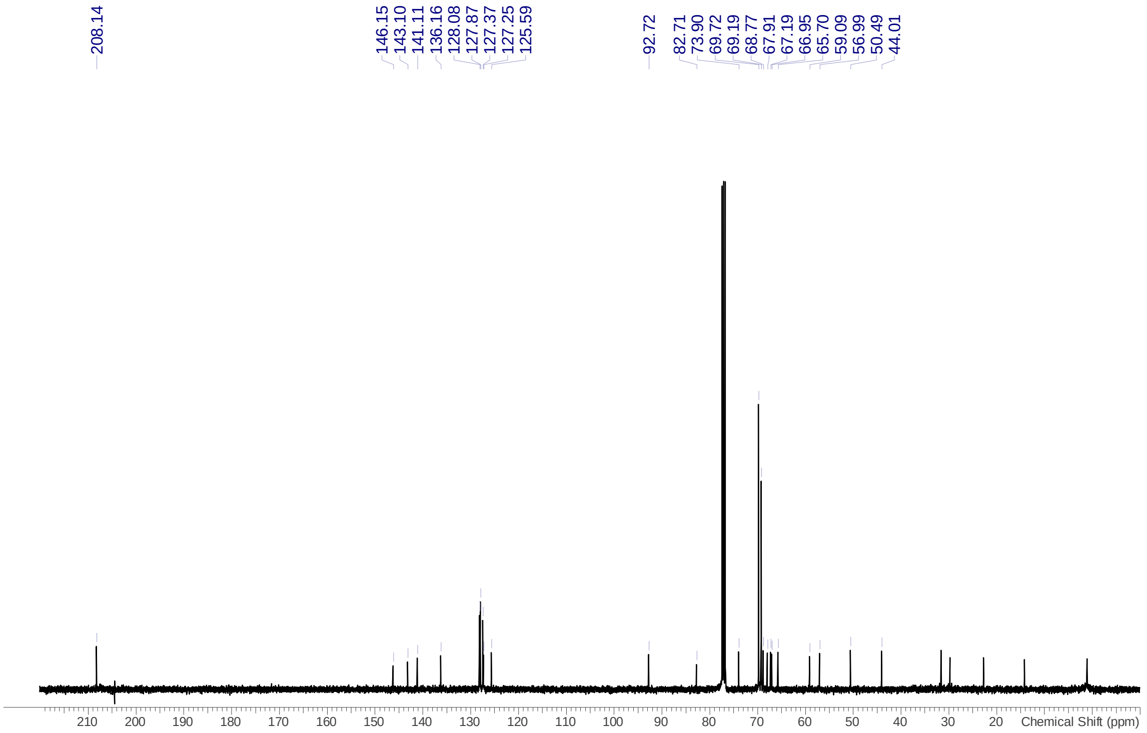

^{13}C NMR spectra ($CDCl_3$, 101 MHz) of **3d** (*n*-hexane in crystals, see elemental analysis).

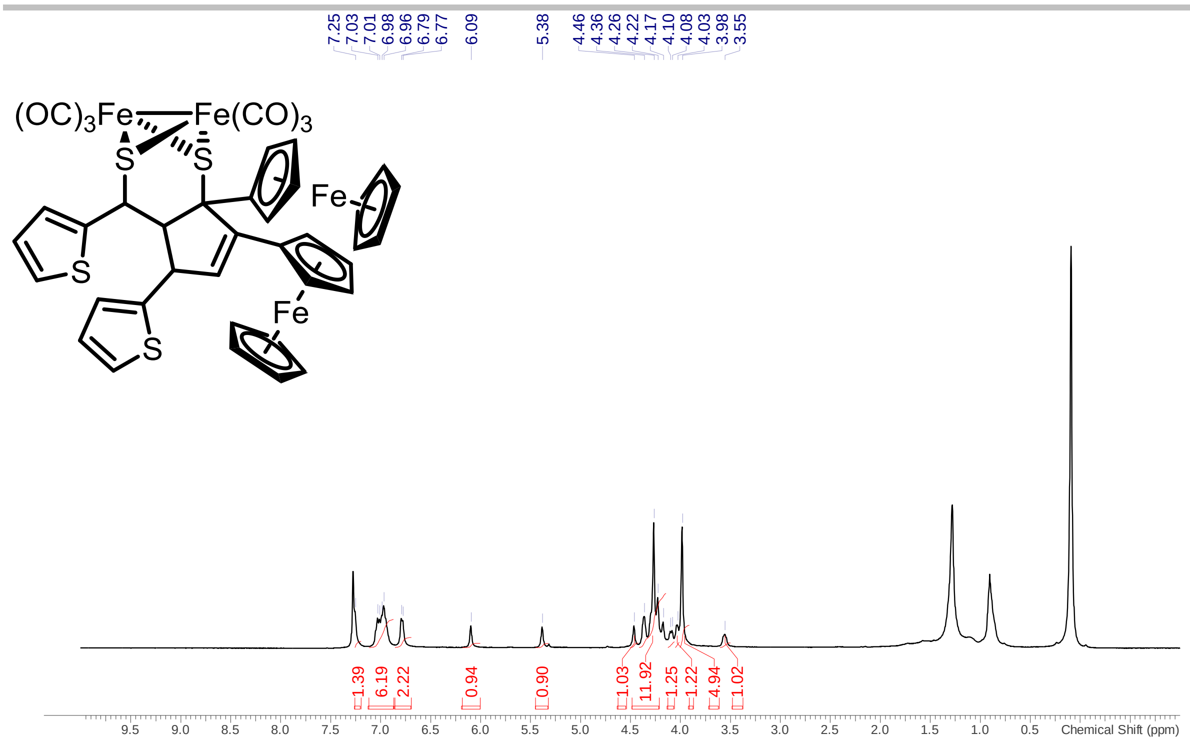

^{1}H NMR spectra ($CDCl_3$, 400 MHz) of **3e** (*n*-hexane in crystals, see elemental analysis).

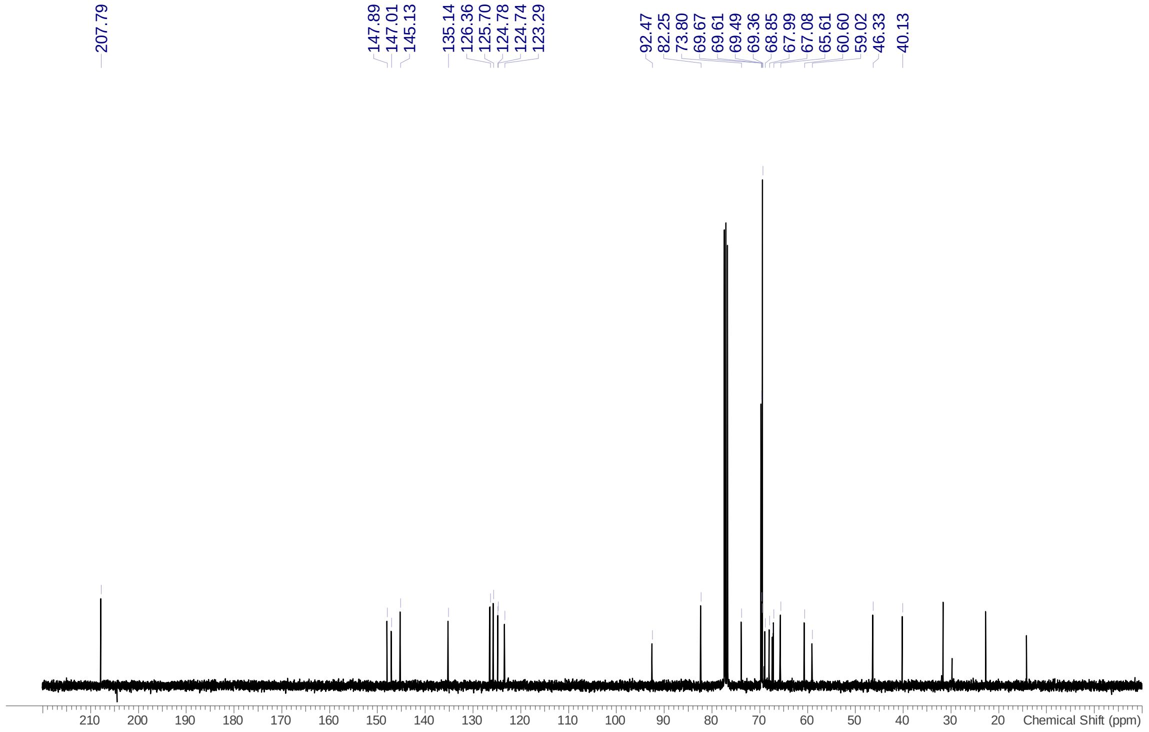

^{13}C NMR spectra ($CDCl_3$, 101 MHz) of **3e** (*n*-hexane in crystals, see elemental analysis).

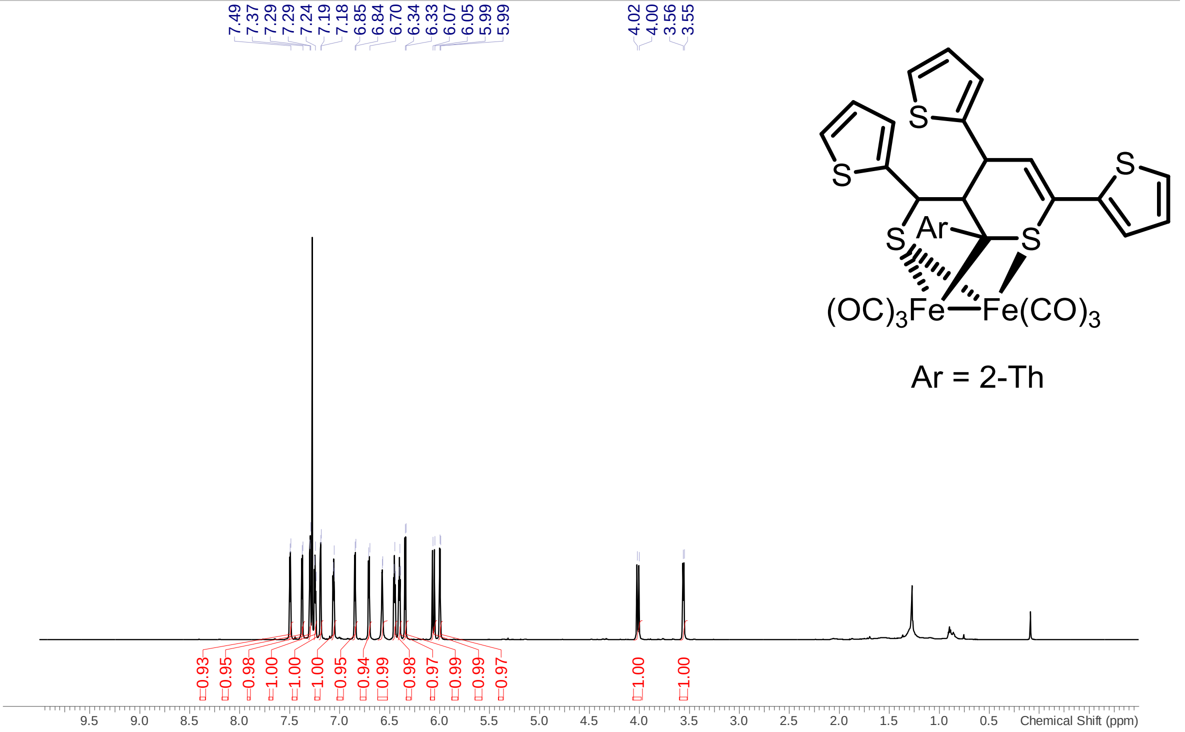

^{1}H NMR spectra ($CDCl_3$, 600 MHz) of **4**.

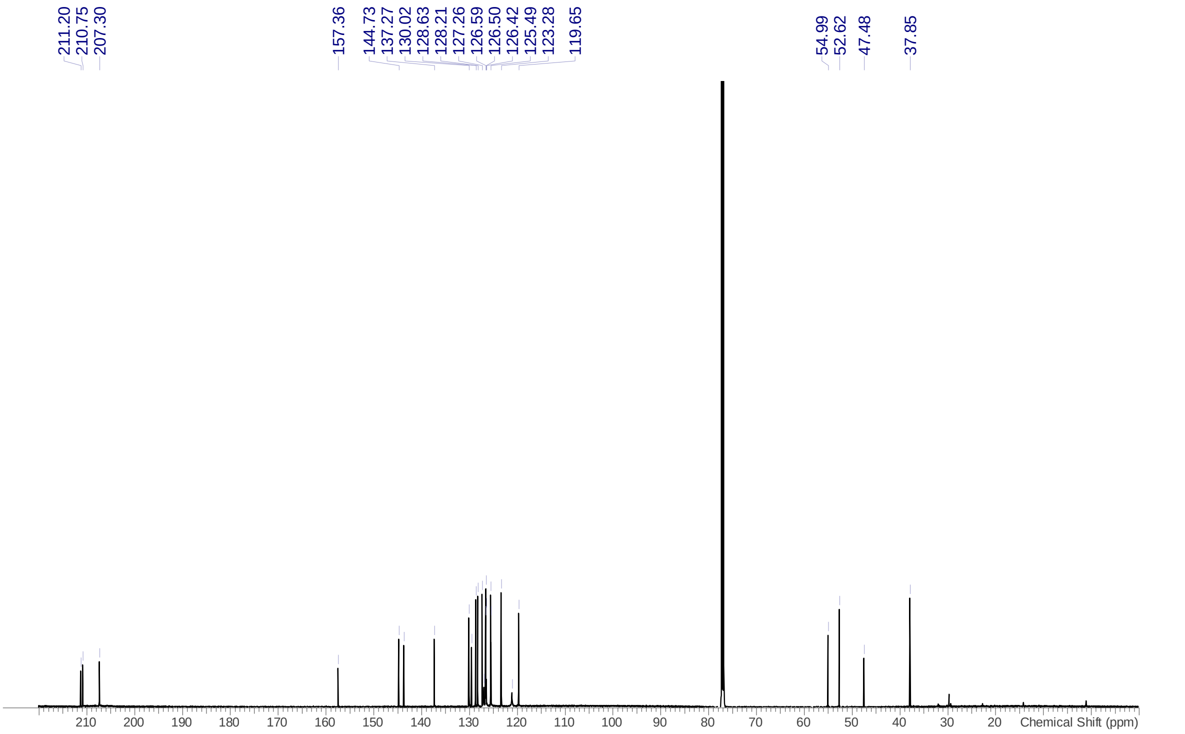

^{13}C NMR spectra ($CDCl_3$, 151 MHz) of **4**.

5 References

[1] a) P. Grzelak, G. Utecht, M. Jasinski, G. Mloston, *Synthesis* **2017**, *49*, 2129; b) G. Mloston, P. Grzelak, H. Heimgartner, *J. Sulfur Chem.* **2017**, *38*, 1; c) G. Mloston, R. Hamera-Faldyga, H. Heimgartner, *J. Sulfur Chem.* **2018**, *39*, 322.
[2] W. McFarlane, G. Wilkinson, *Inorg. Synth.* **1966**, *8*, 181.
[3] H. Alper, D. A. Brandes, *Organometallics* **1991**, *10*, 2457.
[4] a) D. Feller, E. D. Glendening, D. E. Woon, M. W. Feyereisen, *J. Chem. Phys.* **1995**, *103*, 3526; b) M. W. Feyereisen, D. Feller, D. A. Dixon, *J. Phys. Chem.* **1996**, *100*, 2993; c) F. Weigend, M. Häser, *Theor. Chem. Acc.* **1997**, *97*, 331.
[5] a) T. H. Dunning, Jr., *J. Chem. Phys.* **1989**, *90*, 1007; b) D. E. Woon, T. H. Dunning, Jr., *J. Chem. Phys.* **1993**, *98*, 1358.
[6] R. M. Parrish, L. A. Burns, D. G. A. Smith, A. C. Simmonett, A. E. DePrince, E. G. Hohenstein, U. Bozkaya, A. Y. Sokolov, R. Di Remigio, R. M. Richard, J. F. Gonthier, A. M. James, H. R. McAlexander, A. Kumar, M. Saitow, X. Wang, B. P. Pritchard, P. Verma, H. F. Schaefer, K. Patkowski, R. A. King, E. F. Valeev, F. A. Evangelista, J. M. Turney, T. D. Crawford, C. D. Sherrill, *J. Chem. Theory Comput.* **2017**, *13*, 3185.
[7] a) Z. Rolik, M. Kallay, *J. Chem. Phys.* **2011**, *135*, 104111; b) Z. Rolik, L. Szegedy, I. Ladjanszki, B. Ladoczki, M. Kallay, *J. Chem. Phys.* **2013**, *139*, 094105.
[8] a) M. Kallay, P. R. Nagy, D. Mester, Z. Rolik, G. Samu, J. Csontos, J. Csoka, P. B. Szabo, L. Gyevi-Nagy, B. Hegely, I. Ladjanszki, L. Szegedy, B. Ladoczki, K. Petrov, M. Farkas, P. D. Mezei, A. Ganyecz, *J. Chem. Phys.* **2020**, *152*, 074107; b) MRCC, a quantum chemical program suite written by M. Kallay, P. R. Nagy, D. Mester, Z. Rolik, G. Samu, J. Csontos, J. Csoka, P. B. Szabo, L. Gyevi-Nagy, B. Hegely, I. Ladjanszki, L. Szegedy, B. Ladoczki, K. Petrov, M. Farkas, P. D. Mezei, and A. Ganyecz. See www.mrcc.hu.
[9] V. N. Staroverov, G. E. Scuseria, J. Tao, J. P. Perdew, *J. Chem. Phys.* **2003**, *119*, 12129.
[10] F. Weigend, *Phys. Chem. Chem. Phys.* **2006**, *8*, 1057.
[11] a) F. Neese, *Wiley Interdiscip. Rev.: Comput. Mol. Sci.* **2012**, *2*, 73; b) F. Neese, *Wiley Interdiscip. Rev.: Comput. Mol. Sci.* **2017**, *8*, e1327.
[12] F. Neese, F. Wennmohs, A. Hansen, U. Becker, *Chem. Phys.* **2009**, *356*, 98.
[13] a) A. D. Becke, E. R. Johnson, *J. Chem. Phys.* **2005**, *123*, 154101; b) A. D. Becke, E. R. Johnson, *J. Chem. Phys.* **2006**, *124*, 221101; c) E. R. Johnson, A. D. Becke, *J. Chem. Phys.* **2006**, *124*, 174104.
[14] a) G. Henkelman, H. Jonsson, *J. Chem. Phys.* **2000**, *113*, 9978; b) G. Henkelman, B. P. Uberuaga, H. Jonsson, *J. Chem. Phys.* **2000**, *113*, 9901.
[15] A. V. Marenich, C. J. Cramer, D. G. Truhlar, *J. Phys. Chem. B* **2009**, *113*, 6378.
[16] J. Steinmetzer, Pysisyphus, https://doi.org/10.5281/zenodo.3663318, **2020**.
[17] S. Grimme, C. Bannwarth, P. Shushkov, *J. Chem. Theory Comput.* **2017**, *13*, 1989.
[18] a) R. W. W. Hooft, COLLECT, Data Collection Software; Nonius B.V., Netherlands, **1998**; b) L. Krause, R. Herbst-Irmer, G. M. Sheldrick, D. Stalke, *J. Appl. Crystallogr.* **2015**, *48*, 3; c) Z. Otwinowski, W. Minor, in *Methods in Enzymology, Vol. 276*, Academic Press, **1997**, p. 307.
[19] G. M. Sheldrick, *Acta Crystallogr., Sect. A: Found. Crystallogr.* **2008**, *64*, 112.
[20] G. M. Sheldrick, *Acta Crystallogr., Sect. C: Struct. Chem.* **2015**, *71*, 3.
[21] A. L. Spek, *Acta Crystallogr., Sect. C: Struct. Chem.* **2015**, *71*, 9.
[22] a) S. Gao, Y. Liu, Y. Shao, D. Jiang, Q. Duan, *Coord. Chem. Rev.* **2020**, *402*, 213081; b) G. A. N. Felton, C. A. Mebi, B. J. Petro, A. K. Vannucci, D. H. Evans, R. S. Glass, D. L. Lichtenberger, *J. Organomet. Chem.* **2009**, *694*, 2681; c) H. Abul-Futouh, A. Q. Daraosheh, J. Windhager, H. Görls, W. Weigand, *Polyhedron* **2019**, *174*, 114155.
[23] a) G. A. N. Felton, A. K. Vannucci, J. Chen, L. T. Lockett, N. Okumura, B. J. Petro, U. I. Zakai, D. H. Evans, R. S. Glass, D. L. Lichtenberger, *J. Am. Chem. Soc.* **2007**, *129*, 12521; b) S. J. Borg, S. K. Ibrahim, C. J. Pickett, S. P. Best, *C. R. Chim.* **2008**, *11*, 852; c) H. Abul-Futouh, M. El-khateeb, H. Görls, K. J. Asali, W. Weigand, *Dalton Trans.* **2017**, *46*, 2937; d) H. Abul-Futouh, L. R. Almazahreh, M. K. Harb, H. Görls, M. El-khateeb, W. Weigand, *Inorg. Chem.* **2017**, *56*, 10437.
